# Natural and engineered inflammasome adapter proteins reveal optimum linker length for self-assembly

**DOI:** 10.1016/j.jbc.2022.102501

**Published:** 2022-09-16

**Authors:** Pedro Diaz-Parga, Andrea Gould, Eva de Alba

**Affiliations:** 1 Department of Bioengineering, School of Engineering, University of California Merced, California, USA; 2 Quantitative Systems Biology PhD Program, University of California Merced, California, USA

**Keywords:** inflammasome adapter, ASC, protein self-assembly, interdomain dynamics, interdomain linker, NMR relaxation, multidomain protein function, CARD, caspase activation and recruitment domain, DED, death effector domain, DLS, dynamic light scattering, MW, molecular weight, ns-TEM, negative-staining transmission electron microscopy, PYD, pyrin domain, SEC, size-exclusion chromatography, NMR, nuclear magnetic resonance

## Abstract

The inflammasome is a multiprotein complex that triggers the activation of proinflammatory cytokines. The adapter ASC and its isoform ASCb mediate inflammasome assembly *via* self-association and oligomerization with other inflammasome proteins by homotypic interactions of their two identical Death Domains, PYD and CARD, connected by a linker of different length: 23 (ASC) and 4 (ASCb) amino acids long. However, ASC is a more potent inflammasome activator compared to ASCb. Thus, adapter isoforms might be involved in the regulation of the inflammatory response. As previously reported, ASC’s faster and less polydisperse self-association compared to ASCb points to interdomain flexibility resulting from the linker length as a key factor in inflammasome regulation. To test the influence of linker length in self-association, we have engineered the isoform ASC3X with identical PYD and CARD connected by a 69 amino acid-long linker (*i.e*., three-times longer than ASC’s linker). Real-time NMR and dynamic light scattering data indicate that ASC3X polymerization is less effective and more polydisperse compared to ASC or ASCb. However, transmission electron micrographs show that ASC3X can polymerize into filaments. Comparative interdomain dynamics of the three isoforms obtained from NMR relaxation data reveal that ASCb tumbles as a rod, whereas the PYD and CARD of ASC and ASC3X tumble independently with marginally higher interdomain flexibility in ASC3X. Altogether, our data suggest that ASC’s linker length is optimized for self-association by allowing enough flexibility to favor intermolecular homotypic interactions but simultaneously keeping both domains sufficiently close for essential participation in filament formation.

Protein structure and dynamics determine function and operating mode. Specifically, interdomain motion plays a critical role in the function of multidomain proteins. The combination of domains is a frequent strategy to naturally develop new functions using minimal resources (1, 2); therefore, approximately 80% of eukaryotic proteins are predicted to be composed of multiple domains (3). The function and three-dimensional structure of multidomain proteins depend on the concerted action of the different domains (4, 5). Stretches of amino acids that connect protein domains (interdomain linkers) may facilitate interdomain communication (6). Based on the amino acid composition and length, interdomain linkers can adopt transient secondary structures or remain intrinsically disordered, hence conferring different degrees of interdomain flexibility (7). Therefore, the length and structure of the linker have a strong impact on domain separation and orientation, leading to changes in the biological function of multidomain proteins. For instance, it has been reported that linker flexibility or lack thereof has a direct influence on catalytic sites, degree of domain separation, interdomain orientation, and coordinated binding in protein–ligand interactions (4, 7). In some cases, linker flexibility might be required to grant a greater amount of domain mobility, allowing the different domains to function independently of each another. The critical role of interdomain linkers is evidenced by studies showing that changes such as deletions and mutations directly impact the biological function of multidomain proteins (8, 9, 10).

Members of the Death Domain superfamily are ideal examples of multidomain proteins (11). Here, we focus on the Death Domain protein ASC (11, 12, 13, 14), which plays a critical role in the assembly of the multiprotein complex inflammasome that triggers the inflammatory response (14, 15, 16, 17). Inflammasome assembly is mediated by homotypic interactions between multiple copies of three proteins: sensor, adapter ASC, and the effector procaspase-1 (17, 18). Death Domain superfamily members typically carry several caspase activation and recruitment domains (CARDs), pyrin domains (PYDs), and death effector domains (DEDs), which are prevalent in the assembly and activation of inflammatory and apoptotic complexes (11, 17, 19). Inflammasome sensors are generally grouped based on their structural features as NLRs (nucleotide-binding domain leucine-rich repeat containing receptors), ALRs (Absent in melanoma 2-like receptors), or pyrin (20, 21, 22). NLRP3, from the NLR family, is one of the best well-characterized inflammasome sensors that has been implicated in the development of several autoimmune and inflammatory diseases such as multiple sclerosis, type 2 diabetes, atherosclerosis, and cryopyrin-associated periodic syndrome (23).

Inflammasome activation is a tightly controlled process involving two steps: priming and activation (24, 25) (Fig. 1). The priming signal triggers toll-like receptors to initiate the nuclear factor NF-kβ pathway that results in the expression of NLRP3, pro-IL-1β, pro-IL-18, and procaspase-1 (24, 25, 26). Simultaneously, a secondary signal (activation) is required to prompt inflammasome assembly. The secondary signal is triggered by a variety of stimuli such as changes in cellular concentration of K^+^, increase in intracellular Ca^2+^, internalization of flagella through endocytosis, pore-forming toxins, and lysosomal disruption, among others (25, 26, 27, 28). Activation and oligomerization of the NLRP3 sensor leads to the recruitment of the adapter ASC and effector procaspase-1 to the inflammasome complex, which ultimately results in procaspase-1 autocleavage into its active form (Fig. 1). Caspase-1 processes proinflammatory cytokines into their active forms, as well as the protein gasdermin-D into its active N-terminus form (29, 30). Once cleaved, the N-terminus of gasdermin-D self-associates forming large pores in the plasma membrane causing the cell to undergo an inflammatory form of death known as pyroptosis (30, 31, 32, 33), which facilitates the release of mature proinflammatory cytokines into the extracellular matrix.

**Figure 1:**
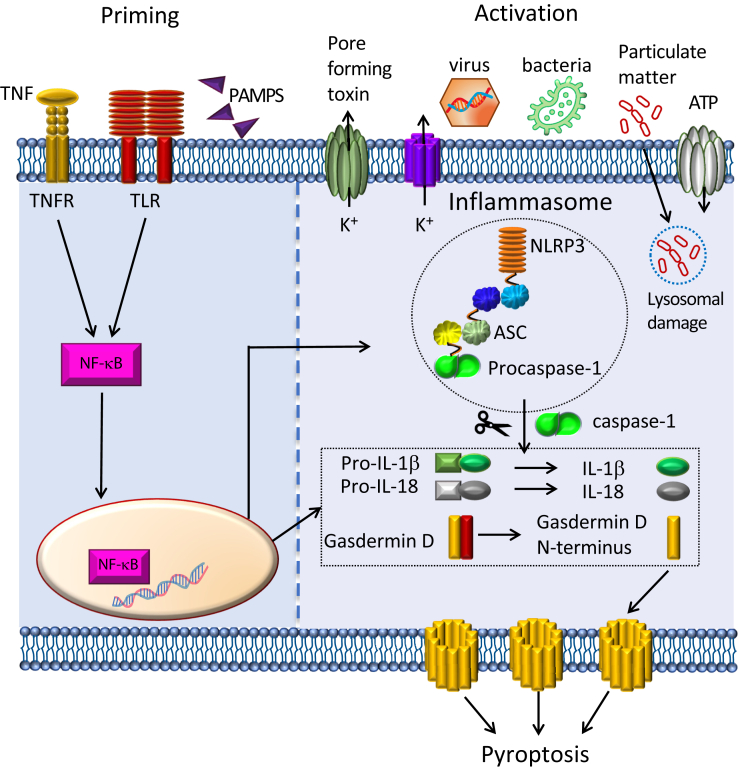
**NLRP3 inflammasome priming and activation.** Priming (*left*) results from the activation of cell receptors by pathogen-associated molecular patterns (PAMPS) and cytokines, leading to the expression of inflammasome components (sensor (NLRP3), ASC, procaspase-1) and cytokine zymogens (pro-IL-1β and pro-IL-18). Activation (*right*) by different stimuli, such as changes in K^+^ efflux, lysosomal damage, or nucleic acids from virus and bacteria, leads to inflammasome assembly and the activation of procaspase-1. Sensor and procaspase-1 are bridged by the adaptor ASC. Upon activation, caspase-1 cleaves proinflammatory cytokines and gasdermin-D. The N-terminus of the latter forms pores in the plasma membrane facilitating cell death by pyroptosis.

ASC functions as a mediator between the sensor and procaspase-1 during inflammasome formation and regulates proper inflammasome assembly (14, 17, 34). ASC is a multidomain protein consisting of a N-terminal PYD and a C-terminal CARD connected by a 23 amino acid linker (14, 21, 35). ASC exists in four different isoforms: canonical ASC, ASCb, ASCc, and ASCd (14). ASC and ASCb are structurally similar, differing only in the length of their linker (23 amino acids *versus* 4 amino acids, respectively) (13, 14). During activation, ASC localizes with the sensor and procaspase-1 forming the inflammasome, which adopts the shape of a micrometer size ring or speck (ASC speck) in the cytosol (36). Colocalization of ASC with caspase-1 and NLRP3 produces a strong inflammatory response measured by IL-1β release to the extracellular milieu (14). In contrast, ASCb is not able to assemble into the typical filamentous ASC speck, instead forming irregular filaments. It has been reported that ASCb is a less potent activator of the inflammasome compared to ASC (14). Interestingly, ASCc, which retains the intact CARD but not the PYD, acts as an inhibitor of the inflammasome complex, whereas the function of ASCd is currently unknown (14).

Previously, we have reported using real-time NMR and dynamic light scattering (DLS) that ASC and ASCb show stark differences in their ability to self-associate (16). We also showed using high resolution NMR that the PYD and CARD of both proteins are identical at the structural level. Thus, differences in self-association must be associated with the length of the linker and with interdomain flexibility. Interdomain dynamics of multidomain proteins can be quantified using ^15^N NMR relaxation techniques to determine local and global dynamics. ^15^N relaxation studies of the protein backbone provide dynamic information that can be correlated to protein function (37). Our previous studies on the structure and dynamics of ASC suggest that interdomain flexibility could facilitate protein–protein interactions by enabling the two domains to act semi-independently of each other, thus allowing each domain to sample a large volume of space without interfering with each other’s binding capability (35).

We report herein the study and comparison of the interdomain dynamics of ASC and ASCb using NMR relaxation techniques. Protein rotational correlation times derived from NMR relaxation data indicate that ASCb tumbles as a rod with little interdomain flexibility, whereas ASC domains are semi-independent. Thus, interdomain flexibility correlates with improved self-association observed for ASC relative to ASCb (16, 35). We hypothesize whether a longer linker would confer additional flexibility and thus better interacting capabilities. To test this hypothesis and further understand the influence of linker length on the function of the inflammasome adapter, we generated an artificial isoform, herein referred to as ASC3X, with a significantly increased linker length (69 amino acids, corresponding to three times the length of ASC linker). The rationale behind the use of a 3X linker is to determine the effect of linker length on interdomain dynamics at one extreme scenario contrasting ASCb’s very short linker. We show by NMR that the PYD and CARD of ASC3X retain the six-helix bundle motif common to death domain proteins. However, real-time NMR data of ASC3X self-association indicate a significantly different behavior compared to ASC and ASCb (16). In addition, DLS studies reveal higher polydispersity compared to the natural isoforms. Size-exclusion chromatographic analysis of ASC3X shows low-order and high-order oligomers, whereas the low-order species was not observed in ASC and ASCb. We reported previously that both PYD and CARD in ASC are integral part of the filament structure. Here, negative-staining transmission electron microscopy (ns-TEM) studies show that ASC3X also assembles into filament bundles with analogous structural characteristics to those formed by ASC and ASCb. Finally, NMR relaxation techniques reveal that interdomain flexibility in ASC3X is only slightly larger than that of ASC. This modest increase suggests that an intermediate size protein such as ASC2X (46 amino acid-long linker) would not lead to significant changes. Altogether, our results point to a critical role played by ASC’s linker length in fast self-association and close to monodisperse oligomerization that involves both interdomain flexibility but also sufficient proximity of the PYD and CARD to form part of the filament structure. In contrast, ASC3X interdomain flexibility is comparable to that of ASC but the two domains are separated by a very long linker, likely slowing down filament and filament bundle formation.

## Results

### ASC3X design and structural comparison with canonical ASC

**Figure 2:**
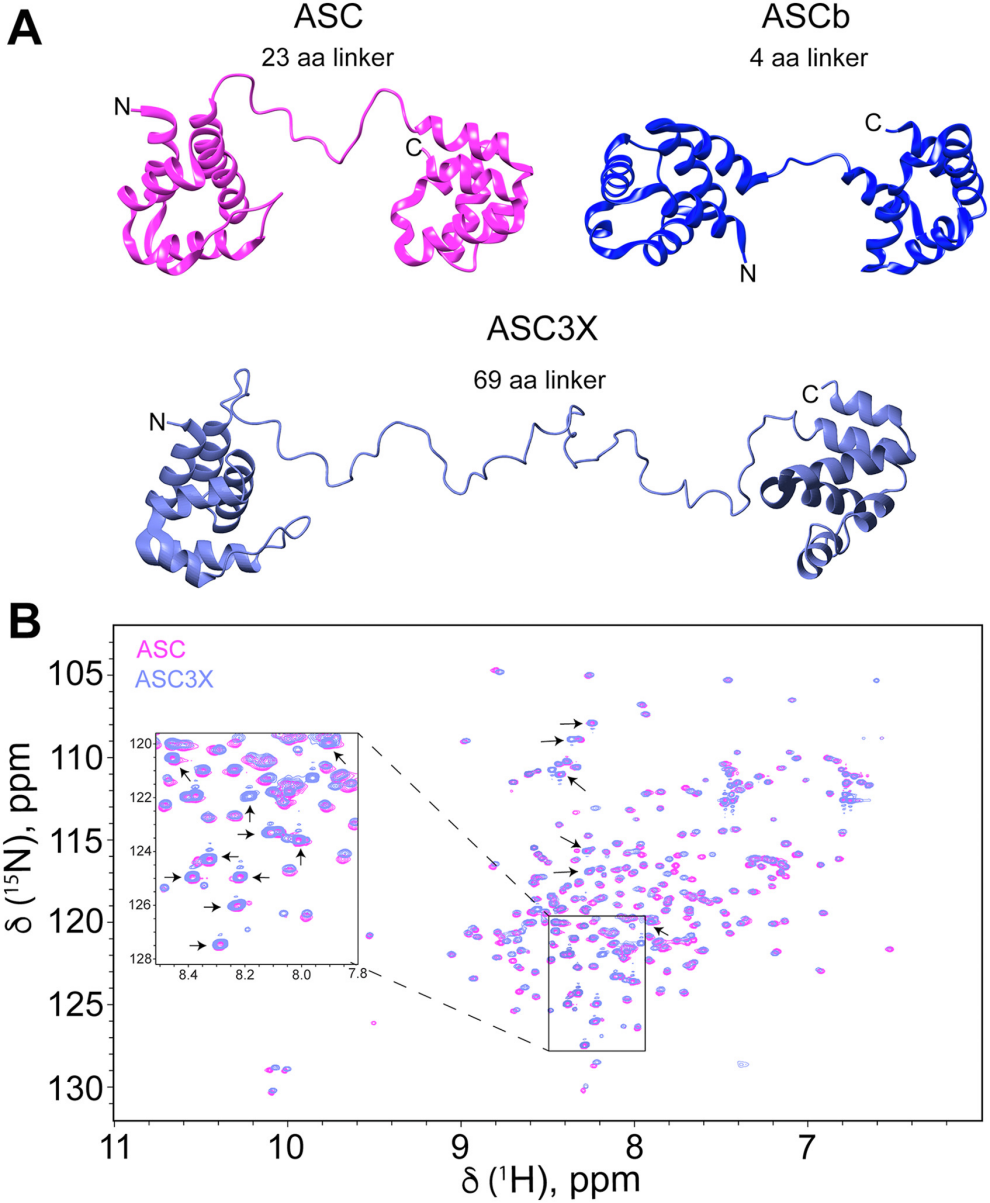
**Structure comparison between ASC, ASCb, and ASC3X.***A*, ribbon diagram of the structures of ASC (35), ASCb (16), and ASC3X (model created with Chimera (57) based on the structure of ASC (35)). *B*, 2D-[^1^H-^15^N]-HSQC: NMR protein fingerprint of ASC and ASC3X showing almost complete signal overlap in the PYD and CARD. A region of the spectrum is zoomed in for clarity. Unassigned peaks of the linker are indicated with *arrows*. “aa” refers to amino acid. CARD, caspase activation and recruitment domain; PYD, pyrin domain.

We reported recently that ASC’s faster and less polydisperse self-association compared to ASCb is related to its longer linker (23 and 4 amino acids for ASC and ASCb, respectively) (amino acid sequences shown in Fig. S1) (16). To test whether a longer linker would further improve the interacting capabilities of ASC isoforms, we engineered an artificial isoform (ASC3X) sharing identical PYD and CARD domains albeit connected by a linker three-times longer than ASC’s linker (Fig. 2*A*) (Amino acid sequence shown in Fig. S1).

We showed previously that ASC and ASCb share the same six-helix bundle motif in the PYD and CARD (16). For an analogous structural comparison between ASC and ASC3X, the NMR chemical shift assignments of ASC3X were inferred from the chemical shifts reported for ASC (38) and checked by triple-resonance NMR. ASC3X ^1^H, ^15^N, ^13^C_α_, and ^13^C_β_ chemical shifts are shown in Table S1 and have been deposited in the Biomagnetic Resonance Bank with ID 51509.

**Figure 3:**
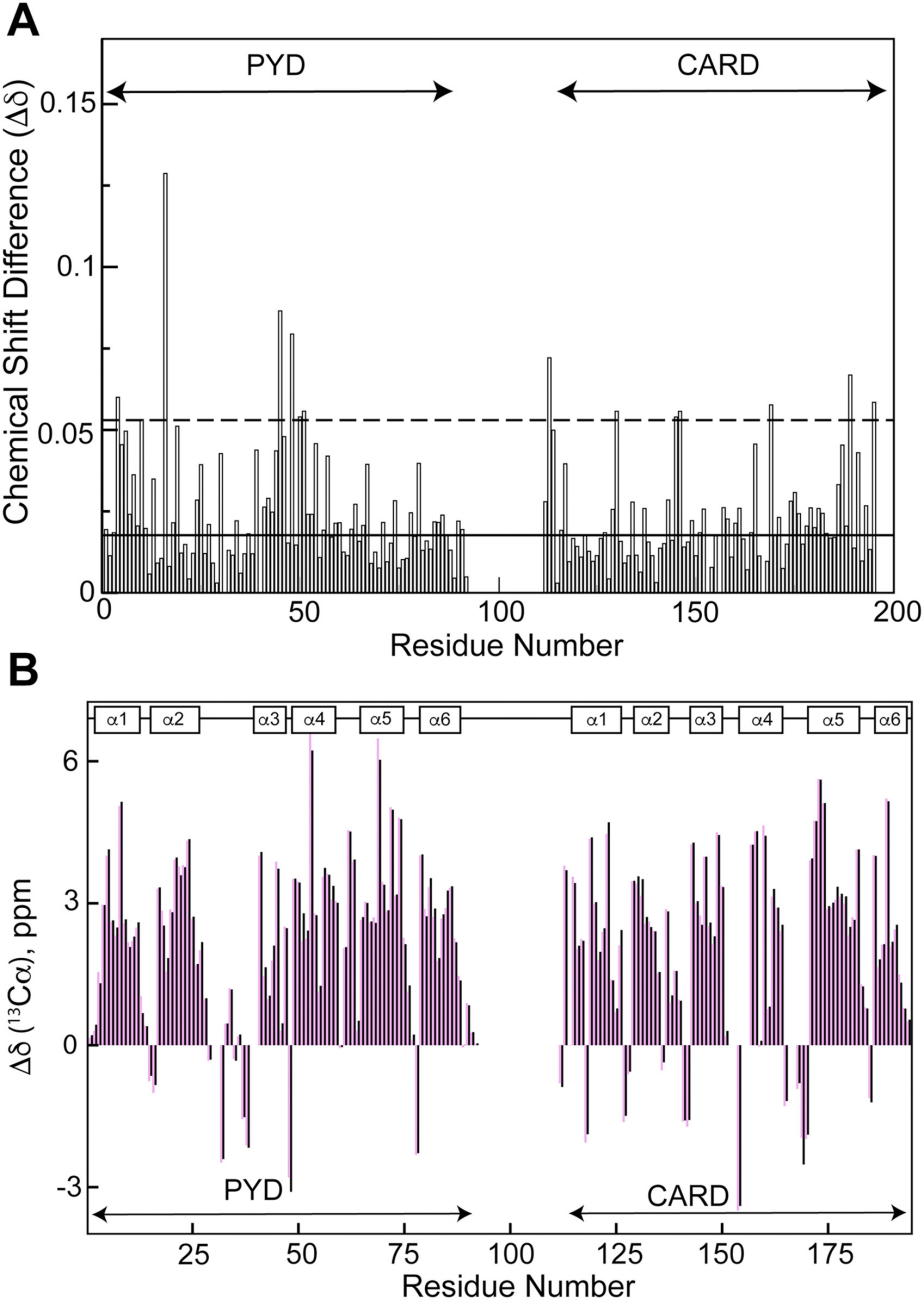
**ASC3X and ASC share equivalent structures of the PYD and CARD domains.***A*, combined ^1^H and ^15^N chemical shift differences between ASC and ASC3X *versus* residue number. (*Solid line*: one SD; *dashed line*: three SD). *B*, secondary shifts of ASC (*pink bars*) and ASC3X (*black bars*), indicating the six helices characteristic of the Death Domain fold. CARD, caspase activation and recruitment domain; PYD, pyrin domain.

The overlay of the amide [^1^H-^15^N]-heteronuclear single quantum coherence (HSQC) spectra of ASC and ASC3X shows signal overlap of most amino acids in the PYD and CARD regions, thus indicating structure equivalence (Fig. 2*B*). The 69 amino acid-long linker in ASC3X results in intense signals in the ^1^H chemical shift interval between 8.0 and 8.5 ppm, which corresponds to amide ^1^H chemical shifts of disordered protein regions (39). Combined amide ^1^H-^15^N chemical shift deviations between ASC and ASC3X are small with a SD value lower than 0.025 ppm (Fig. 3*A*). Larger deviations are expected for residues close to the linker in the amino acid sequence or in the 3D structure. In addition, the ^13^C_α_ chemical shift differences from tabulated random coil values indicate the presence of two six-helix bundle motifs for the PYD and CARD of ASC3X as previously observed for ASC (Fig. 3*B*).

### Different oligomerization kinetics of ASC3X compared to ASC and ASCb by real-time NMR

ASC and ASCb have a strong tendency to polymerize forming filaments and filament bundles under physiological conditions in accord with their function as inflammasome adapters (14, 15, 16). In addition to pH and protein concentration (35), other factors such as temperature and the presence of salt were identified as important determinants in ASC’s oligomerization based on analytical centrifugation (40) and size-exclusion chromatography (SEC) (16).

Upon ASC and ASCb oligomerization, NMR signal intensity decreases with time because large protein assemblies are formed and due to their slow tumbling rates do not contribute to signal intensity (16). NMR signal intensity follows an exponential decay as protein self-association progresses. An analogous effect has also been observed in amyloid β-peptide and prion protein oligomerization (41, 42). Previously, we found that ASC oligomerizes following two kinetic phases. In contrast, a single slower kinetic phase was observed for ASCb. Because the only difference between ASC and ASCb is the length of the linker, we could expect that ASC3X behaves like ASC.

**Figure 4:**
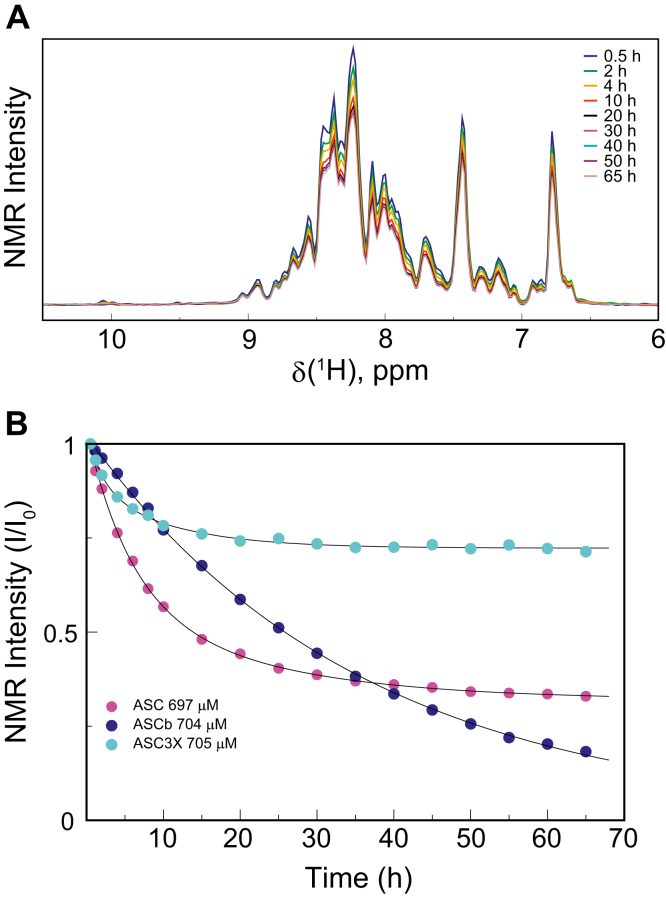
**Less effective oligomerization of ASC3X compared to ASC and ASCb.***A*, overall decay of NMR signal intensity from 1D projections of 2D spectra as a function of time for the oligomerization of ASC3X. *B*, normalized overall NMR signal intensity decay *versus* time for ASC3X, ASC, and ASCb (16) at approximately 700 μM protein concentration.

To study ASC3X oligomerization by real-time NMR, we have followed the same protocol used for ASC and ASCb (16). Briefly, pure ^15^N-labeled protein in lyophilized form is dissolved in the NMR buffer at a concentration of ∼700 μM to start the oligomerization process. A series of 2D-[^1^H,^15^N]-NMR spectra were acquired as a function of time for 65 h. The overall signal intensity of the spectra decreases with time due to oligomerization (Fig. 4*A*). The decrease of NMR signal intensity in ASC3X is analogous to that found in ASC (16), although to a significantly lesser extent, and different from ASCb behavior (Fig. 4*B*). Apparent steady state conditions are reached approximately 20 h after the start of ASC3X oligomerization resulting in a plateau at ∼75% of the original intensity.

Single- and double-exponential equations (Equations 1 and 2, respectively) were used to fit the intensity decay. The fitting to a double-exponential equation was better, which suggests that a minimum of two phases with different rate constants take place in ASC3X oligomerization. Fast and slow rate constants for ASC3X were obtained: *k*_1ASC3X_ = 0.5 h^−1^ and k_2ASC3X_ = 0.09 h^−1^, respectively (Table 1). This result can be explained by protein assembly into different types of oligomers at different rates. The *k*_1_ and *k*_2_ values indicate that the initial decay is faster compared to ASC and ASCb; however, polymerization is less effective (plateau reached at ∼75% of the original intensity). These results suggest that initial ASC3X oligomerized species form fast, but subsequent polymerization to higher order oligomers is not as efficient.

(1) $$\text{y}={\text{A}}_{0}{\text{e}}^{-\text{x k}}+{\text{A}}_{1}$$

(2) $$\text{y}={\text{A}}_{0}{\text{e}}^{-{\text{x k}}_{1}}+\;{\text{A}}_{2}{\text{e}}^{-{\text{x k}}_{2}}+{\text{A}}_{1}$$

**Table 1:** Kinetic parameters from exponential equations 1 and 2

Rate (h^−1^)	Protein isoform		
	ASC3X^a^	ASC^b^	ASCb^c^
k_1_	0.503 ± 0.007	0.15 ± 0.03	0.02 ± 0.01
k_2_	0.097 ± 0.007	0.04 ± 0.01	0.02 ± 0.01
k_1avePYD_	0.30 ± 0.14	0.21 ± 0.04	0.0269 ± 0.0009
k_2avePYD_	0.03 ± 0.02	0.05 ± 0.02	0.0269 ± 0.0009
k_1aveCARD_	0.33 ± 0.06	0.12 ± 0.02	0.0288 ± 0.0009
k_2aveCARD_	0.05 ± 0.03	0.02 ± 0.01	0.0288 ± 0.0009
Goodness of fit			
χ2	0.00044850	0.00032796	0.00059583
r	0.998312	0.999812	0.999806

a: reported error from fitting. b,c: retrieved from ref (16).

By performing this analysis at the amino acid level, we reported previously that the PYD and CARD domains of ASC show different behavior in the first kinetic phase with *k*_1_ values of the PYD larger compared to the CARD (Table 1). However, the *k*_2_ values are similar for both domains. This behavior was not observed for ASCb (Table 1). Our results led us to suggest that PYD-PYD interactions drive the first step in the oligomerization reaction of ASC, followed by PYD and CARD participation in subsequent polymerization steps. In contrast, equivalent participation of both domains is apparent in the polymerization reaction of ASCb.

**Figure 5:**
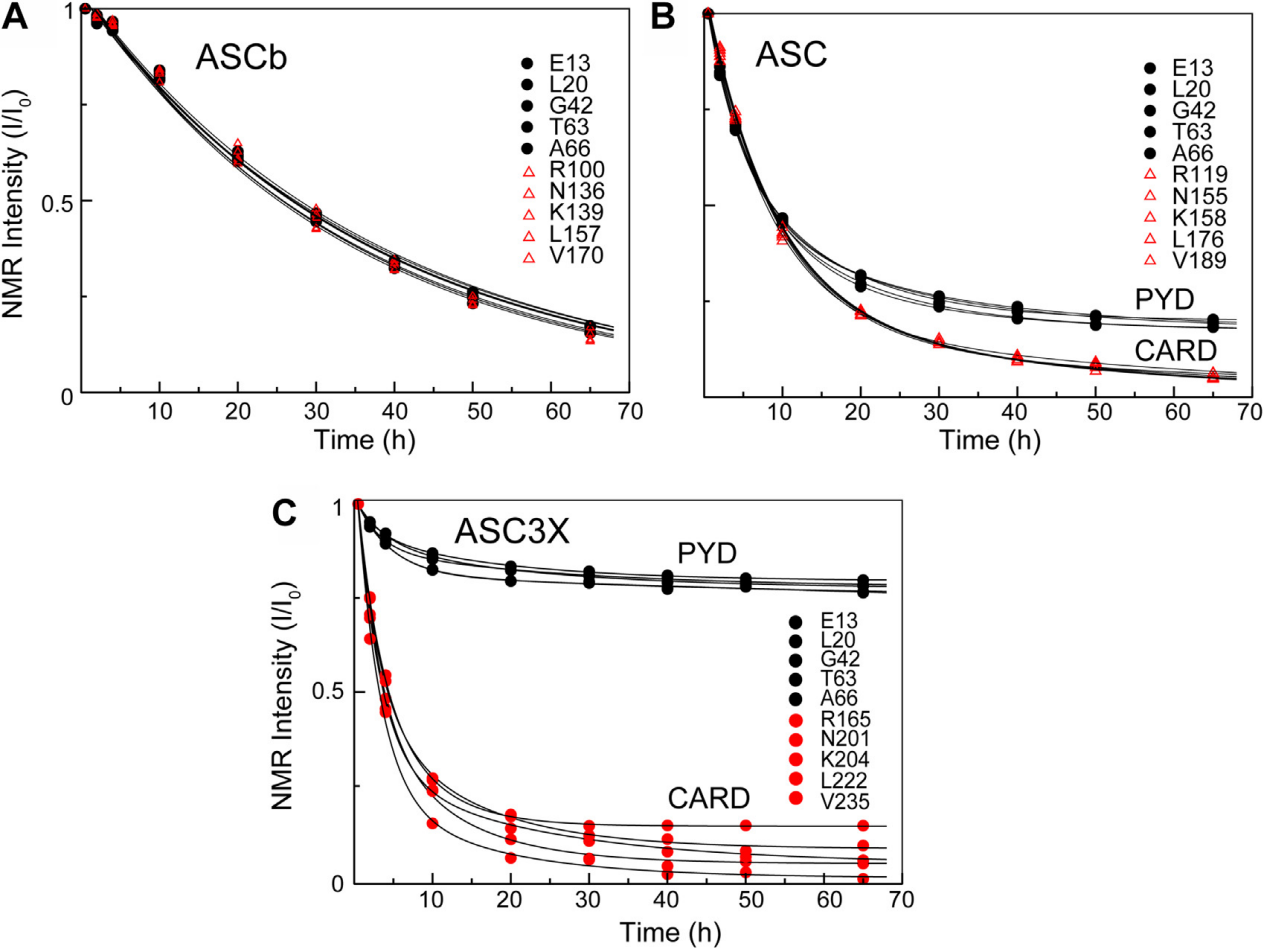
**The PYD and CARD domains display larger differences in self-assembly kinetics as the linker length increases.***A*, NMR signal intensity decay for selected amino acids in the PYD and CARD domain of ASCb (*A*), ASC (*B*), and ASC3X (*C*). The helix to which each amino acid belongs is indicated in the main text. CARD, caspase activation and recruitment domain; PYD, pyrin domain.

**Figure 6:**
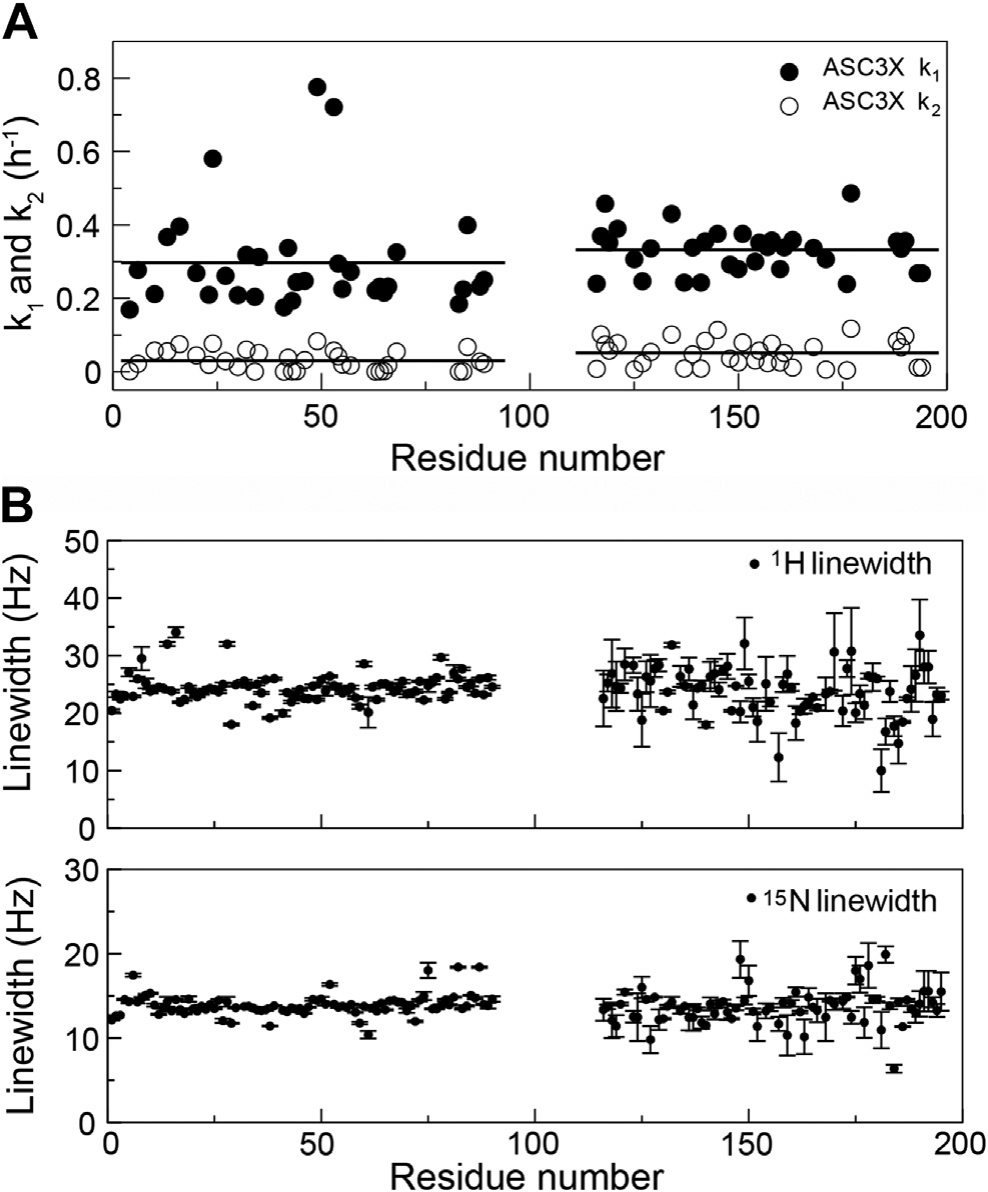
**Real-time NMR kinetics of ASC3X self-assembly reveals an important role of the CARD domain.***A*, rate constants (*k*_1_ and *k*_2_) of 33 signals from the PYD and 33 signals from the CARD (from five amino acids per helix and three amino acids in the loop connecting helices 2 and 3) *versus* residue number. *Solid black lines* are averages obtained for each dataset. *B*, differences in linewidth (at half-height) are average values for all time points for the PYD and time points up to 10 h for the CARD for ^1^H (*top*) and ^15^N (*bottom*) amide signals in 2D-[^1^H-^15^N]-HSQC spectra. Error bars are SDs. CARD, caspase activation and recruitment domain; PYD, pyrin domain.

For ASC3X, we selected amino acids giving rise to well-resolved NMR signals and representative of the protein secondary structure. Specifically, we selected residues in the PYD from helices 1 (E13), 2 (L20), 3 (G42), and 5 (T63 and A66) and in the CARD from helices 1 (R119), 4 (N155, K158), 5 (L176), and 6 (V189) (numbering follows ASC sequence, Fig. S1). The fitting of NMR signal intensity decay of the individual amino acids to the exponential equations reveals that the PYD and CARD follow different behaviors (Fig. 5). The average rate constants (*k*_1_ and *k*_2_) of 33 analyzed signals from each domain (five residues in each of the six helices and three amino acids in the long loop connecting helices 2 and 3) are similar (Fig. 6*A* and Table 1); however, the intensity of CARD signals decreases to values close to zero (∼6%), whereas PYD signals show much smaller decrease to values ∼76% of the initial intensity (Fig. 5). In addition, *k*_1_ and *k*_2_ rate constants for individual amino acids in PYD and CARD are smaller than the values obtained for the overall intensity decay when all amino acids are included (Table 1). This discrepancy could be attributed to contributions of the long linker to signal intensity and to the significantly different kinetic behavior of the PYD and CARD.

The substantial decrease in CARD signal intensity could be explained by its participation in homotypic interactions resulting in the formation of initial oligomeric species in which the PYD is not involved. These first oligomers will have highly mobile PYDs connected to oligomerized CARDs by the long linkers. The higher mobility will result in intense signals from amino acids in the PYDs. To further investigate the abrupt intensity decay in CARD signals, we have monitored amide signal linewidth for both ^1^H and ^15^N in the of 2D-[^1^H-^15^N]-HSQC spectra for all time points for the PYD and only up to 10 h after the start of the experiment for the CARD because signal intensity for the latter is close to baseline level after 10 h (Fig. 6*B*). The average signal width at half height is similar for both domains (Fig. 6*B*). However, while there is almost no variability in signal width with time for the PYD, significant variations (∼5 Hz) are observed for the CARD. This variability might reflect a larger error in the CARD due to the abrupt loss in signal intensity, as well as an effect of the kinetics of CARD oligomerization in signal broadening. This effect occurs when the exchange rate (interconversion rate) between monomer and initial oligomeric species is similar in magnitude to the difference in resonance frequency for CARD nuclei in the different species. This behavior was not observed for ASC and ASCb oligomerization and indicates that ASC3X self-association follows different kinetics and possibly the formation of initial species with different half-lives.

We have shown previously that both PYD and CARD are integral part of the ASC filament (15). In ASC, the two domains are connected by a linker sufficiently long to allow for reorientation, thus favoring multiple interactions, but also sufficiently short for the readily participation of the PYD and CARD in filament formation (15). In contrast, the participation of both domains in filament formation for ASC3X could be hindered by the large separation between the two domains. Overall, real-time NMR results indicate that the engineered isoform follows different kinetics for oligomerization compared to the natural isoforms. For ASC3X, the CARD domain appears to be the initial driving force of the polymerization, followed by the PYD. Based on the low intensity decay of the PYD in ASC3X compared to the PYD in ASC (16), the oligomerization capabilities of the engineered isoform have diminished, suggesting that an interdomain linker longer than the natural one does not enhance polymerization but is in fact disruptive.

To test whether chemical modifications influence the kinetics of ASC3X self-association, we used mass spectrometry on protein samples prepared several weeks after oligomerization started. Using liquid chromatography (reverse phase) coupled to mass spectrometry, the ASC3X spectrum shows a single peak that corresponds to the monomeric species (Fig. S2*A*). The molecular weight from mass spectrometry (28,204.9 g/mol) matches the theoretical molecular weight of monomeric ^15^N-labeled ASC3X (28,202.9 g/mol), thus ruling out chemical modifications during self-association. The oligomerized ASC3X sample was also studied by mass spectrometry avoiding the unfolding step of the reverse phase column. In this case, both monomer and dimer (molecular weight (MW) of the dimer is 56,399.5 g/mol) were observed (Fig. S2*B*). This result suggests that ASC3X forms stable dimers.

### ASC3X forms oligomers of different sizes detected by SEC

SEC was used to study oligomer size distribution of ASC3X at different conditions of polymerization: time, protein concentration, and pH. We reported previously using NMR and analytical ultracentrifugation that NaCl and high protein concentration promotes ASC self-association (40). Protein samples for the chromatographic analysis contain 150 mM NaCl to avoid nonspecific interactions with the matrix. The samples were not filtered to allow detection of large size oligomers but were centrifuged for 1 min to pellet down potential precipitated material.

**Figure 7:**
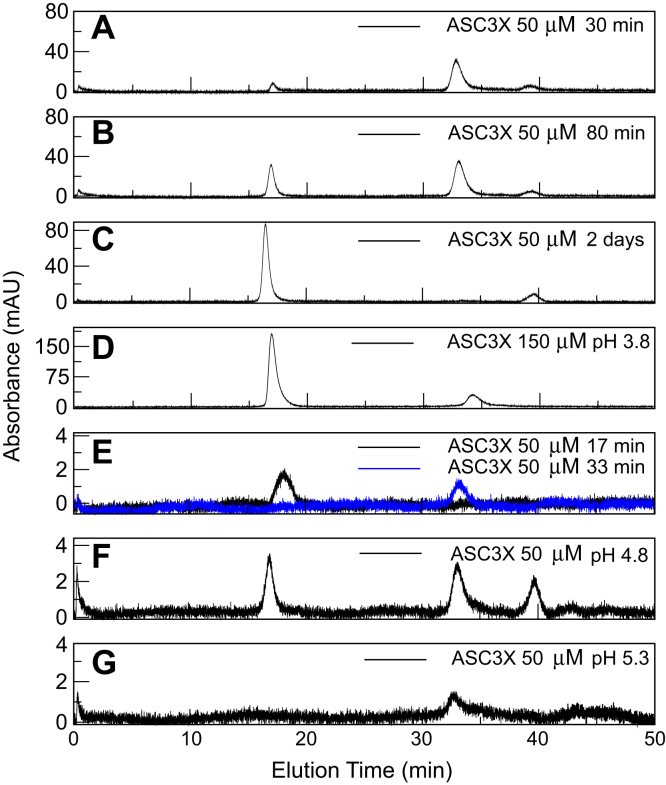
**ASC3X oligomer size distribution by size-exclusion chromatography.***A*–*C*, chromatograms of ASC3X at 30 min, 80 min, and 2 days after sample preparation (50 μM ASC3X, pH 3.8). *D*, 30 min after sample preparation (150 μM ASC3X, pH 3.8). *E*, overlay of chromatograms resulting from the injection of eluted solutions at 33 and 17 min from chromatogram in (*A*). *F* and *G*, 30 min after sample preparation (50 μM ASC3X, pH 4.8 and 5.3).

ASC3X samples at 50 μM concentration and pH 3.8 were subjected to SEC (Superdex 200 10/300 GL: molecular weight range of 10–600 kDa) for 30 min, 80 min, and 2 days after preparation. The resulting chromatograms show absorbance peaks (at 280 nm) at elution times of ∼17, ∼33, and ∼39 min for samples at 30 and 80 min after sample preparation (Fig. 7, *A* and *B*). The first elution peak matches the void volume of the column, thus corresponding to oligomers with MW >600 kDa. According to the SEC calibration and considering a globular protein, ASC3X dimer and monomer should elute at ∼29 min and ∼32 min, respectively. However, ASC3X has a very long linker and thus increased retention times are expected, as this effect has been reported for ASC (16). Because retention times of 33 and 39 min are significantly different than the expected values, we cannot derive conclusions on the oligomeric state of the protein corresponding to each peak. However, these different oligomeric states were not observed for ASC or ASCb. An SDS-PAGE analysis of the peaks at 33 and 39 min shows monomer and dimer for the former and monomer only for the latter (Fig. S3). This result, together with the mass spectrometry data (Fig. S2*B*), suggests that ASC3X forms stable dimers and is indicative that the solutions eluted at 33 and 39 min correspond to different oligomeric species.

Overall, ASC3X behavior differs from that of ASC and ASCb in SEC studies, as the former assembles into long-lived oligomers of different size, whereas only oligomers > 600 kDa and monomeric species were observed for the latter (16). The peak at 33 min in the ASC3X chromatogram is absent in the chromatogram obtained 2 days after sample preparation, and only the peaks at 17 and 39 min of elution time remain (Fig. 7*C*). This result indicates that the species at 33 min finally disappears with time to form higher order oligomers. We also tested the influence of protein concentration. At higher protein concentration (150 μM), the peak at 39 min is absent from the chromatogram (Fig. 7*D*) and the oligomer at 33 min is observed. This result suggests that the peak at 39 min is likely a low-order oligomer (or a monomer) that further oligomerizes and that the peak at 33 min is stable and favored at higher protein concentration. To test the reversibility of the oligomerization process, the peaks at 17 min (high-order oligomers) and 33 min (long-lived, low-order oligomer) were collected and reinjected in the SEC column. The resulting chromatograms show peaks at the same elution times (Fig. 7*E*). In the latter chromatograms, the signal to noise ratio has decreased due to protein dilution during the SEC process. This result suggests that once the low-order or high-order oligomers are formed, the equilibrium is significantly shifted toward these species. The data in Figure 7*E* corroborate the high stability of the oligomer at 33 min inferred from its presence in the chromatograms shown in (*A*), (*B*), and (*D*).

To investigate by SEC the effect of pH in the oligomerization of ASC3X, we acquired chromatograms at pH 4.8 and 5.3. The protein precipitates significantly by pH increase. However, the chromatogram at pH 4.8 resulting from the supernatant after centrifugation shows three peaks at elution times equivalent to those observed at pH 3.8 (Fig. 7*F*). As expected, the three peaks show lower absorbance values because of protein mass lost upon precipitation. In contrast, at pH 5.3, only the peak corresponding to the low-order oligomer at 33 min remains (Fig. 7*G*), suggesting that the high-order oligomers, possibly more populated and larger, fall out of solution. The presence of the peak at the void volume indicates that ASC3X oligomers are >600 kDa, thus corresponding to a lower limit of ∼21 ASC3X protomers.

### ASC3X polymerizes forming filaments of similar width compared to ASC and ASCb

The analysis of ASC and ASCb macrostructures using ns-TEM has been reported previously (15, 16). Both proteins readily form filaments with an average width of ∼ 7 nm (15, 16) frequently stacked forming bundles. Bundles composed of three and four filaments are more abundant for ASC, whereas two-filament bundles are prominent in ASCb. Bundle width is a multiple of the width of the individual filament and matches the number of filaments in the bundle, thus indicating that filaments align laterally. ASC filaments are formed by stacked rings with the size of an ASC dimer, suggesting a possible mechanism for filament growth (15).

**Figure 8:**
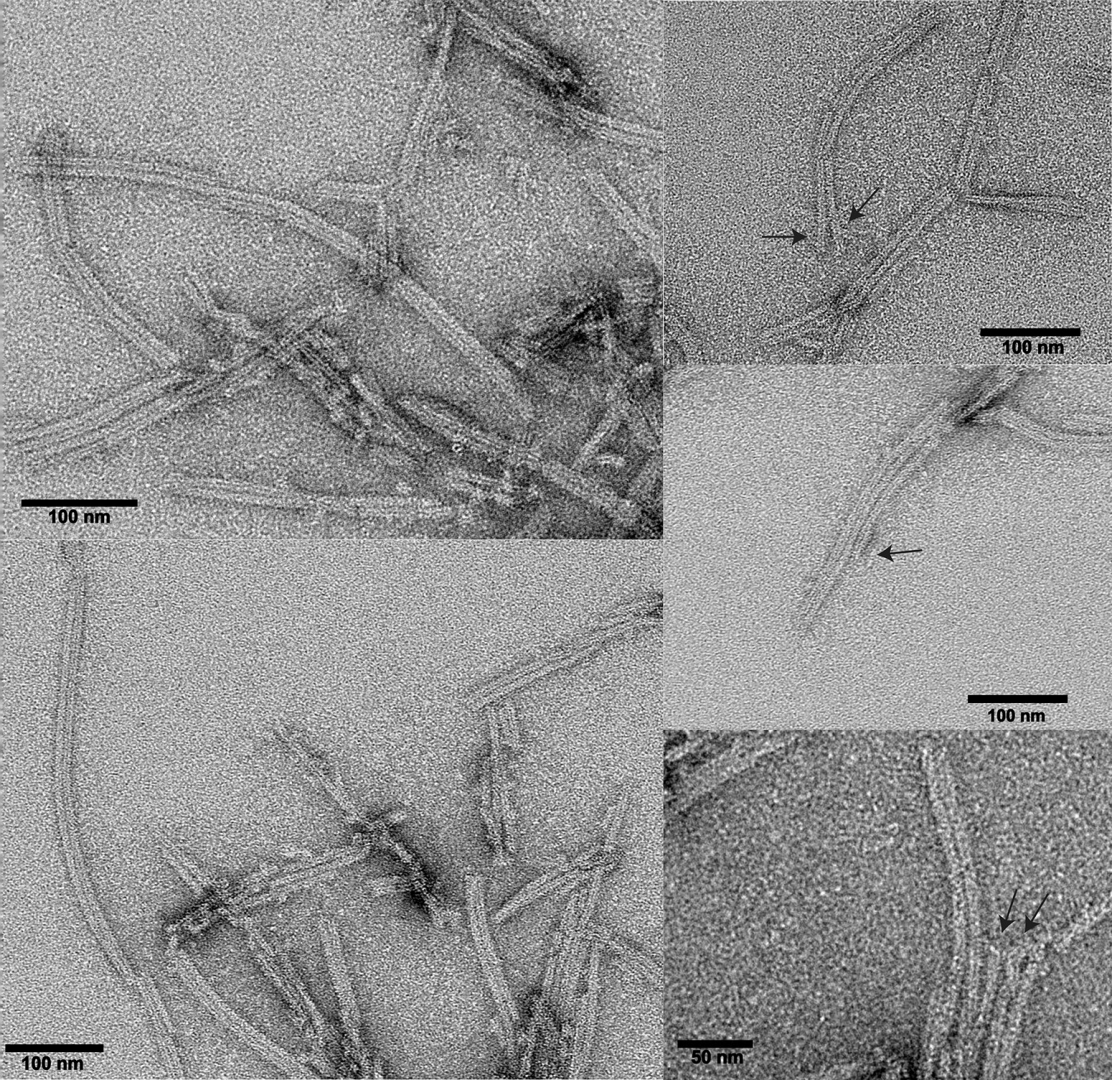
**ASC self-assembles predominantly into two-filament bundles.** ns-TEM micrographs showing ASC3X bundles at different magnification. *Arrows* point to the separation of a two-filament bundle into individual, constituent filaments (*up*), and infrequent three-filament bundles (*bottom*). ns-TEM, negative-staining transmission electron microscopy.

An analogous analysis by ns-TEM indicates that ASC3X has high propensity of forming 2-filament bundles (Fig. 8). In contrast to ASC and ASCb, individual, isolated filaments were not observed for ASC3X and three-filament bundles were very infrequent. An average bundle width of 15.19 ± 0.53 nm was measured based on the analysis of 50 ASC3X filament bundles composed of two filaments. The width of individual filaments obtained from the 2-filament bundles is 6.94 ± 0.22 nm. These results indicate that despite the very long linker, ASC3X can form filaments of similar width to those formed by ASC and ASCb. By combining the real-time NMR and ns-TEM results, we can conclude that the long linker in ASC3X modifies the oligomerization kinetics but not the basic capability to polymerize forming filaments. The analysis of multiple macrostructures clearly shows a predominance of 2-filament bundles; exceptionally few three-filament bundles were observed (Fig. 8). Therefore, additional lateral stacking of filaments forming multiple-filament bundles is hindered in ASC3X.

It is worth to note that the experimental conditions used for filament formation (TEM) and real-time NMR experiments are different. Neutral pH is needed in the former and acidic pH is required in the latter to shift oligomerization equilibria toward the monomeric species that leads to appreciable NMR signal. The ns-TEM analysis shows that the less effective oligomerization of ASC3X is partially overcome at neutral pH. However, the lack of multiple-filament bundles in ASC3X compared to ASC suggests that additional oligomerization is hindered in the former. At this point we can only speculate whether ASC3X long linker is responsible for the preponderance of two-filament bundles (almost exclusively present) and the lack of individual filaments and bundles with multiple (>2) filaments.

### ASC3X self-association is more polydisperse compared to ASC and ASCb

**Figure 9:**
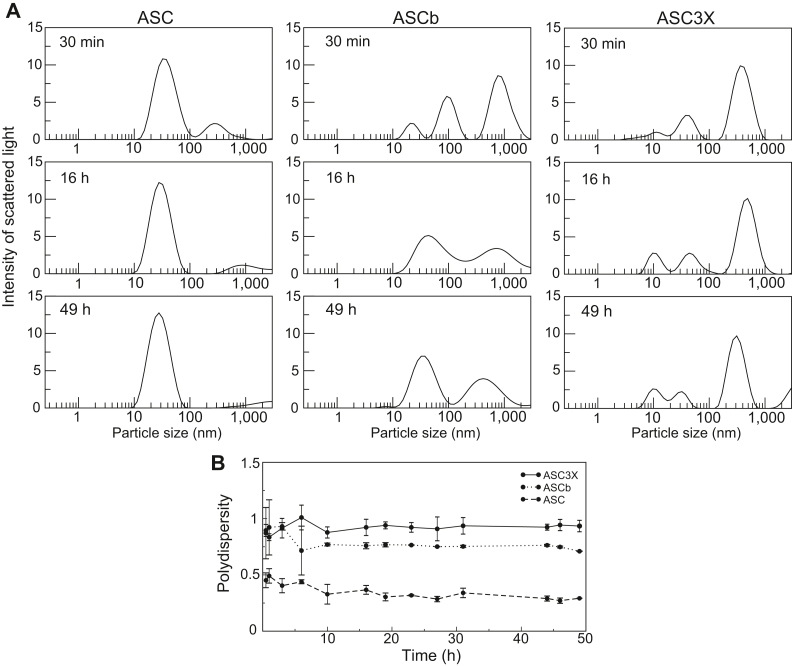
**Time-dependent population distribution of ASC, ASCb, and ASC3X oligomers by dynamic light scattering.***A*, changes over time in intensity of scattered *light versus* size of ASC, ASCb (16) and ASC3X oligomers. *B*, changes in polydispersity values of ASC, ASCb (16) and ASC3X solutions during the oligomerization process. Data in (*A* and *B*) are an average of three measurements and error bars in (*B*) represent SD. The 30 min time point can be considered as 0 min time point after sample preparation.

DLS was used to study the kinetics of ASC3X oligomerization. Figure 9 shows how the intensity of the scattered light *versus* particle size changes during 49 h of polymerization. The intensity of the scattered signal varies with the population and size of the different species present in solution. The dimensions shown in Figure 9 do not correspond to the real oligomer size because ASC3X oligomers are filamentous (Fig. 8), and a perfect sphere is assumed in the calculation of particle size. Nonetheless, the DLS data allow observing populations of species with various assembly sizes and how they change as self-assembly proceeds.

According to the DLS data, ASC3X forms species categorized in three group sizes, ∼10, ∼40, and ∼400 nm, 30 min after sample preparation, which is the time required to obtain the first DLS measurements (Fig. 9*A*). As oligomerization progresses, small variations in the population and size of these species are observed, reaching steady state at 49 h with peaks at ∼10, ∼30, and ∼300 nm. The ASC3X species detected by real-time NMR could correspond to the peak at ∼10 nm, with high-order oligomers represented by the other two peaks. As oligomerization proceeds, the peaks at ∼40 and ∼400 nm decrease in size, suggesting that the oligomers are more close packed. At the end of the kinetic experiment, ASC3X still shows three species of different size, whereas ASC showed only one species and ASCb showed two (Fig. 9*A*) (16). Importantly, the overall polydispersity value (p) of the ASC3X solution at the end of the polymerization experiment is fairly constant and close to 1 (p_ASC3X_ = 0.93 ± 0.05) (Fig. 9*B*), contrasting with the lower polydispersity values of ASC and ASCb (p_ASC_ = 0.35 ± 0.04 and p_ASCb_ = 0.79 ± 0.06) (16).

Previously reported DLS data on ASC and ASCb polymerization agree with the former forming a speck of uniform size and the latter polymerizing into clustered and disordered filaments inside the cell (14, 16). A behavior closer to that of ASC was expected for ASC3X. However, polymerization of the artificial isoform leads to the largest polydispersity. Altogether, results from cell assays, real-time NMR, and DLS suggest that the length of ASC linker is optimal for faster polymerization leading to solutions of lower polydispersity. In addition, ASC shows higher tendency to form multiple filament bundles based on ns-TEM data (15), which might be required for the formation of ASC filamentous ring-specks with a rim approximately 150 nm wide (36). We may attribute the slower polymerization kinetics, higher polydisperse oligomers, and less densely packed macrostructures of ASCb and ASC3X to the very short and very long linkers of the former and latter, respectively.

### Interdomain dynamics in ASC isoforms increases with linker length

Recently, we have shown using molecular docking that the proximity of the PYD and CARD in ASCb, due to the short linker, hinders its interactions with the CARD of procaspase-1 (16). This result was not observed for ASC with two domains sufficiently separated by the much longer linker (16). In addition, we showed based on previous NMR relaxation studies that the two domains in ASC have different rotational correlation times due to the flexibility of the linker (35). We have performed analogous studies to investigate the influence of the linker length in the interdomain dynamics of the natural and designed isoforms, ASCb and ASC3X, respectively.

**Figure 10:**
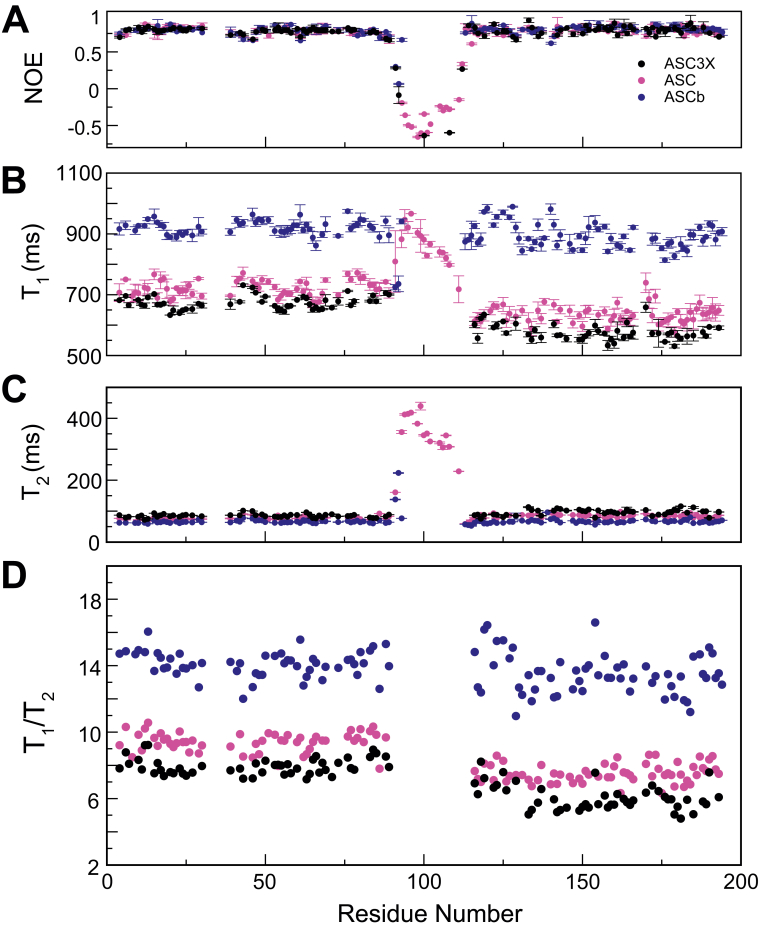
**Interdomain dynamics in ASC3X, ASC, and ASCb from**^**15**^**N amide NMR relaxation.***A*, heteronuclear NOE. *B*, longitudinal (T_1_), (*C*) and transverse (T_2_) relaxation times. *D*, T_1_/T_2_ ratios of ASC (*magenta*), ASCb (*blue*), and ASC3X (*black*).

Information on the NMR relaxation properties of the protein backbone amide (^15^N) was obtained by measuring the heteronuclear Overhauser effect ([^1^H-^15^N]-NOE), as well as the longitudinal (T_1_) and transverse (T_2_) relaxation times for the three isoforms (Fig. 10). These three parameters depend on the amide ^15^N-H bond dynamics and the rotational diffusion of the molecule (37, 43). Information on backbone dynamics is readily inferred from heteronuclear NOE experiments as residues adopting regular secondary structure show NOE values in the 0.78 to 0.83 range, compared to values lower than ∼0.65 for residues located in more flexible regions such as linkers and loops (44, 45). For better comparison, these studies have been performed for the three proteins, ASC, ASCb, and ASC3X, under the same experimental conditions, including same magnet, and using identical NMR pulse programs designed to avoid water interference that results in increased measurement errors (46).

The average heteronuclear NOE values for the PYD and CARD regions in ASC3X, ASC, and ASCb are the values expected for two rigid structures (Table 2 and Fig. 10*A*). In contrast, these values start to decrease in the regions close to the PYD C-terminus and the CARD N-terminus toward the linker center (Fig. 10*A*). The very long linker in ASC3X shows NMR signal overlap and chemical shifts could only be assigned for a few amino acids; however, the trend toward low or negative heteronuclear NOE values in this region is clear. These results indicate that the linker undergoes local motions on a fast time scale compared to molecular tumbling. The heteronuclear NOE data indicate that ASC, ASCb, and ASC3X comprise two well-ordered, rigid domains.

**Table 2:** Average ^15^N relaxation parameters for ASC isoforms

^15^N relaxation parameter	Protein isoform					
	ASC3X		ASC		ASCb	
	PYD	CARD	PYD	CARD	PYD	CARD
T_1_ (ms)	674 ± 5	577 ± 12	722 ± 14	639 ± 19	925 ± 13	898 ± 13
T_2_ (ms)	83.4 ± 0.3	94.2 ± 0.7	76.9 ± 0.6	83.6 ± 0.7	65.5 ± 0.3	66.7 ± 0.5
NOE	0.78 ± 0.02	0.78 ± 0.03	0.79 ± 0.03	0.79 ± 0.03	0.79 ± 0.02	0.81 ± 0.02
T_1_/T_2_	7.9 ± 0.5	6.0 ± 0.8	9.4 ± 0.6	7.5 ± 0.6	14.1 ± 0.8	13 ± 1

Clear differences are observed in the average T_1_ values for ASCb compared to ASC and ASC3X (Table 2 and Fig. 10*B*). If interdomain motion is not considered and we assume globular shape for the isoforms, the T_1_ values should decrease concomitantly with a decrease in the protein MW. Thus, the largest T_1_ values would correspond to ASC3X (highest MW) and the lowest values to ASCb (lowest MW). We observe the opposite: average T_1_ values are largest for ASCb and lowest for ASC3X. This result is a consequence of the non-globular shape and responds to the presence of interdomain dynamics. As interdomain mobility increases, the NMR relaxation parameters approximate to values expected for the individual domains. Thus, we suggest two extreme models for interdomain dynamics of the three isoforms: (1) each domain is dynamically independent and (2) both domains tumble as a single rigid body.

We can expect significant interdomain flexibility in ASC3X due to the 69 amino acid-long linker. Thus, T_1_ values for both PYD and CARD are the smallest (Table 2), as the two domains could almost be considered independent from one another. T_1_ values for PYD (MW = 9,951.5 Da) are ∼100 ms larger than for the CARD (MW = 9,737.1 Da) in part because of the larger MW of the former compared to the latter. This result reflects the dynamic independence of one domain from the other. An analogous situation is found for ASC, with the 23 amino acid-long linker also conferring interdomain flexibility. The T_1_ values in ASC are slightly higher compared to ASC3X (Table 2) because the PYD and CARD feel the drag from each other more pronouncedly. The shorter linker in ASC results in slightly less interdomain flexibility. The T_1_ values for the PYD are also larger than for the CARD in ASC, but the difference between the average values is ∼80 ms. The decrease in the difference between T_1_ values is again a consequence of ASC's shorter linker. In contrast, NMR relaxation results for ASCb are very different. The linker connecting the PYD and CARD of ASCb is only four amino acids long, and therefore, we expect a great reduction in interdomain flexibility. In fact, the average T_1_ values for ASCb are the largest out of the three isoforms even though its MW is the smallest. In this case, the average T_1_ value for the PYD is larger than that of the CARD but the difference is only ∼27 ms. This behavior can only be explained by largely diminished interdomain dynamics, which results in both domains tumbling almost as a rigid body.

The differences in T_2_ values between the three isoforms are not as large as for T_1_, but appreciable (Fig. 10*C* and Table 2), and follow the expected trend matching the reduction of interdomain dynamics for the shorter linkers. Thus, we observe higher average T_2_ values for ASC3X and lower for ASCb. However, based on the MW, we would expect the opposite result for globular proteins, which again shows the impact of interdomain dynamics on the NMR relaxation data. In addition, T_2_ values are smaller for the PYD than for the CARD of ASC3X and ASC, indicating significant interdomain flexibility with a trend toward the expected results for independent domains. Furthermore, the difference in T_2_ values for the PYD and CARD is larger for ASC3X than for ASC due to the additional flexibility. In contrast, this difference is much smaller for the PYD and CARD of ASCb, which indicates that the two domains tumble together. The different T_2_ values of the PYD and CARD could also result from differences in the stability of the folded domains.

To obtain more information on the degree of interdomain dynamics, we analyzed amide ^15^N T_1_/T_2_ ratios that are similar for domains tumbling together and different otherwise (44). The ^15^N T_1_/T_2_ ratios of the PYD and CARD of ASC and ASC3X are noticeably different, indicating that they reorient at different rates. In contrast, these values are more similar for the PYD and CARD of ASCb, suggesting that both domains tumble at similar rates (Fig. 10*D* and Table 2). No significant differences are obtained in T_1_/T_2_ ratios for the PYD and CARD of ASC3X and ASC (∼1.9 for both proteins), which suggests that the very long linker in ASC3X confers only marginally additional interdomain flexibility.

To further investigate differences in interdomain dynamics, we have obtained rotational correlation times (τ_c_) for the full-length isoforms and the individual PYD and CARD domains by applying the Lipari-Szabo model-free formalism to our NMR relaxation data (Table 3) (45, 47). As a reference for comparison, we show in Table 3 the experimental and theoretical τ_c_ values obtained for the PYD-only protein ASC2 (48) with similar size and structure to ASC individual domains, and for the N- and C-terminal domains of the protein calmodulin (44).

**Table 3:** Experimental and theoretical rotational correlation times (τ_c_) for ASC3X, ASC, and ASCb

Isoform	Experimental τ_c_ (ns)	Theoretical τ_c_ (ns)^a^
Full length ASC3X	*7.6* ± *0.1*	*16.8 (241 residues)*
ASC3X^PYD^	**8.3 ± 0.2**	**6.7**
ASC3X^CARD^	**7.1 ± 0.2**	**6.2**
Full length ASC	*8.6* ± *0.4*	*13.8 (195 residues)*
ASC^PYD^	**9.2 ± 0.3**	**6.7**
ASC^CARD^	**8.1 ± 0.3**	**6.2**
Full length ASCb	*11.4* ± *0.2*	*12.5 (176 residues)*
ASCb^PYD^	**11.6 ± 0.2**	**6.7**
ASCb^CARD^	**11.3 ± 0.2**	**6.2**
ASC2^b^	*6.2*	*6.3 (84 residues)*
Calmodulin (N-terminal)^b^	*7.1*	*4.1 (73 residues)*
Calmodulin (C-terminal)^b^	*6.3*	*3.7 (66 residues)*

a obtained with a spherical model (65) using the number of residues specified and the corresponding temperature: ASC3X, ASC and ASCb (298 K), ASC2 (298 K), and calmodulin (308 K).

b reported experimental values for ASC2 (48) and calmodulin (44).

The NMR-derived τ_c_ values of both PYD and CARD in ASC3X and ASC (highlighted in bold in Table 3) are only slightly larger than the experimental τ_c_ value of ASC2, indicating that the two domains are independent at the dynamic level for both isoforms. These results suggest that the PYD and CARD dynamic behavior in ASC and ASC3X is closer to the extreme model in which each domain is dynamically independent. The slightly larger values for ASC and ASC3X compared to ASC2 indicate the dragging effect of the linker. This effect is more pronounced for ASC, resulting in larger differences with respect to ASC2, due to a shorter linker compared to ASC3X. In addition, we observe larger τ_c_ values for the PYD relative to the CARD, which matches the expected slower tumbling of the former due to the higher MW of the PYD. In contrast, τ_c_ values of the PYD and CARD in ASCb are significantly larger than the experimental values for ASC2. Moreover, the difference in τ_c_ values between the PYD and CARD in ASCb is very small and close to the experimental error. Both results indicate that ASCb dynamic behavior matches the model where both domains tumble as a rigid body. Interestingly, the experimental and theoretical τ_c_ values (based on molecular size) for the full-length proteins (highlighted in italics in Table 3) are very similar for ASCb (with a difference of ∼1 ns) and disparate for ASC and ASC3X, reaching a difference of ∼9 ns for the latter. These data point again to the independent dynamics of the PYD and CARD in ASC and ASC3X and lack thereof in ASCb.

It has been reported that interdomain motion resulting from linker flexibility has important roles in protein function (4, 7). A well-known example is the multifunctional protein calmodulin with two domains connected by a relatively long linker (15 amino acids) that promiscuously binds to multiple partners (44, 49). NMR relaxation studies on calmodulin show that interdomain flexibility facilitates domain reorientations and simultaneous interactions of both domains with the different ligands (44, 50). Analogously to calmodulin, ASC and its isoform ASCb also interact with different inflammasome proteins (caspase-1 and sensor) and self-associate for inflammasome assembly. Each domain in ASC and ASCb bind to different molecules *via* homotypic interactions. Importantly, the NMR-derived τ_c_ values for the N- and C-terminal domains in calmodulin are significantly larger than the theoretical values (44) (Table 3), thus agreeing with the results obtained for ASC and ASC3X. Proteins within the Death Domain superfamily, such as FADD, have been reported to behave like ASCb at the dynamic level with domains tumbling together (51).

### Discussion: ASC linker length is optimal for self-assembly

ASC and ASCb show differences in inflammasome activity as the former results in higher levels of released IL-1β than the latter (14). In addition, ASC forms the regular speck in the cytosol of activated macrophages, whereas ASCb forms filamentous polymers (14). These results suggest that ASC isoforms could impact the regulation of the inflammatory response by differential inflammasome activation. ASC and ASCb self-association are key in inflammasome assembly. Recently, we have studied the self-association capabilities of ASC and ASCb using several biophysical and biochemical techniques (16). Our studies show that ASC polymerizes faster and forms less polydisperse oligomers compared to ASCb, thus explaining at the molecular level ASC’s more efficient inflammasome activation (16). We have suggested that ASC’s long linker provides the flexibility needed for the multiple interactions required in filament formation. In contrast, the short linker in ASCb obstructs its participation in intermolecular interactions.

We hypothesize here whether a longer linker could confer additional flexibility, thus enhancing isoform self-association. To test this hypothesis, we have engineered an artificial adaptor with a linker three times as long as ASC’s linker (ASC3X). Our real-time NMR data indicate that ASC3X shows less effective polymerization resulting in lower intensity decrease of the overall NMR signal compared to ASC and ASCb. Importantly, pronounced differences in NMR data were observed between the PYD and CARD in ASC3X that were absent in ASCb and minimal in ASC. Specifically, NMR signals of the CARD domain decrease significantly, reaching values close to the baseline level, whereas PYD signals diminish less than 25%.

**Figure 11:**
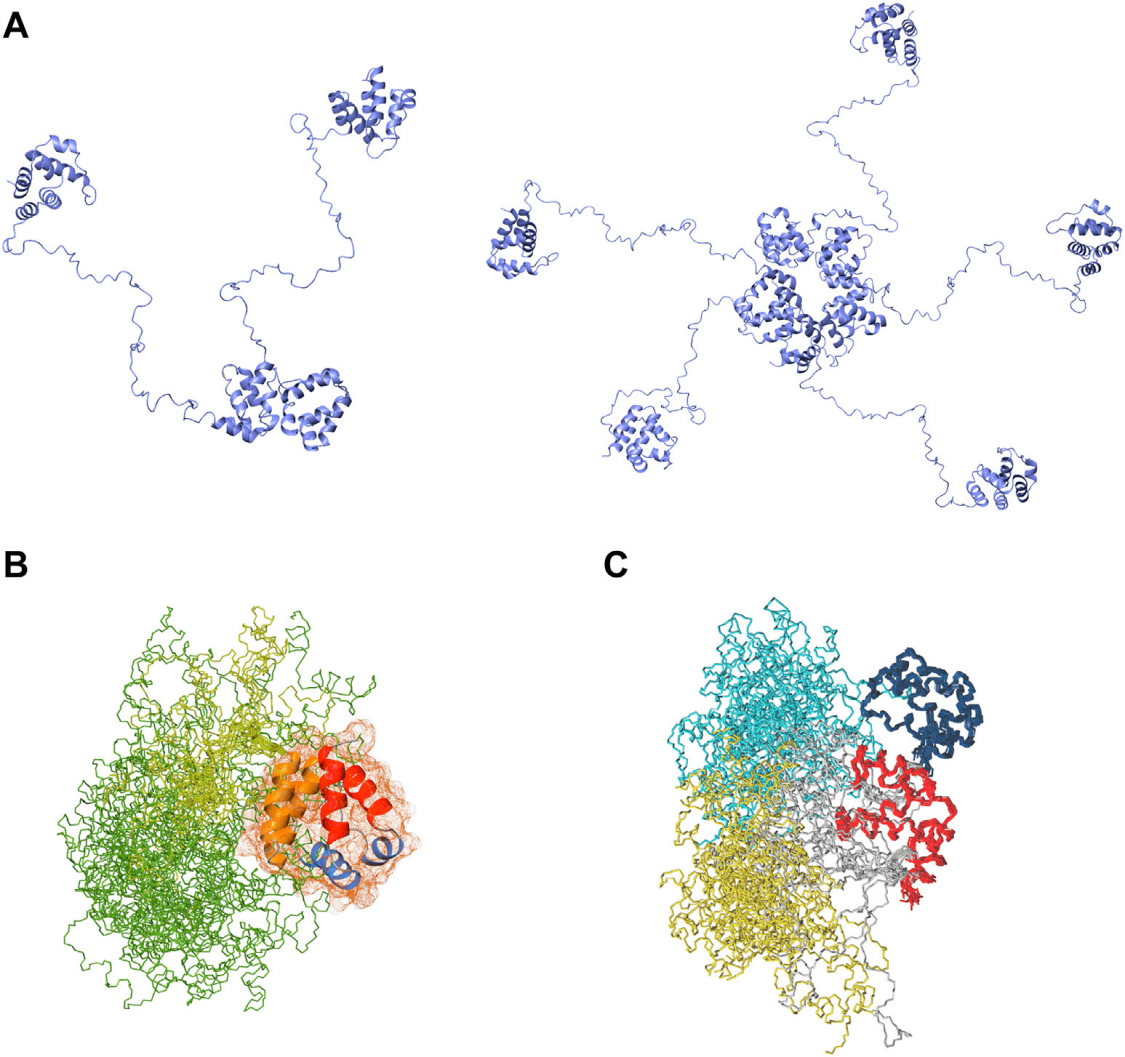
**Molecular models of the self-association of ASC3X and ASC.***A*, models derived from molecular docking of an ASC3X dimer (*left*) and a 5-member oligomer (*right*). *B*, experimental three-dimensional structure of an ASC monomer showing interdomain flexibility (retrieved from ref (35)). *C*, ASC dimer model formed by CARD-CARD binding, showing the proximity of the two free PYDs for additional interactions and dimer stabilization. CARD, caspase activation and recruitment domain; PYD, pyrin domain.

The NMR results suggest that ASC3X assembles into CARD-driven oligomers bearing free (unbound) PYDs at the initial stages of self-association. To illustrate this behavior, we have used molecular docking (52) to create models of ASC3X oligomeric structures *via* CARD–CARD interactions. The molecular docking is driven by the well-known type I interactions between Death Domains, which involve contacts between helices 1 and 4 of one CARD and helices 2 and 3 the other interacting CARD (53, 54, 55, 56). The smallest oligomer is a dimer to which additional ASC3X molecules are added by docking, resulting in a pentameric structure that closes the circle of interacting CARDs (Fig. 11*A*). The molecular models show that the free PYDs are far away from one another to form PYD–PYD interactions that are essential for filament formation (Fig. 11*A*). In contrast, the shorter linker in ASC (Fig. 11*B*) results in a dimer model formed by CARD–CARD interactions that positions the PYDs nearby for homotypic binding (Fig. 11*C*).

Altogether, the real-time NMR data indicate that ASC3X polymerization is initially trapped into oligomers formed with little participation of the PYD. In contrast, both domains show similar participation in ASC and ASCb self-association leading to substantially more effective polymerization. The DLS data show that ASC3X forms oligomers with significantly higher polydispersity compared to ASC and ASCb (Fig. 9*B*), indicating that the very long linker perturbs the oligomerization capabilities. In addition, SEC results show the presence of long-lived ASC3X, low-order oligomeric species that were not observed in analogous experiments for ASC or ASCb. In contrast, when the pH is close to neutral value, ASC3X assembles into filaments of similar dimensions to those formed by ASC and ASCb. Most ASC3X macrostructures are two-filament bundles, which differs from ASC’s tendency to form multiple-filament bundles.

Finally, NMR relaxation data indicate that the PYD and CARD domains in ASCb tumble at very similar rate, showing almost identical τ_c_ values for both domains. In contrast, ASC and ASC3X show PYD and CARD domains tumbling at different rates. However, ASC3X shows marginally larger interdomain flexibility reported by the shorter T_1_ and longer T_2_ values compared to ASC. Overall, NMR relaxation data indicate that ASCb tumbles as a rod with no appreciable interdomain flexibility due to the short linker, whereas ASC and ASC3X domains are very flexible. Interdomain dynamics in both proteins are similar; however, ASC3X self-association is less effective and oligomer polydispersity is larger. To explain this behavior, we need to consider the role that interdomain flexibility and linker length can play in filament formation.

We reported previously that both PYD and CARD are integral components of filament assembly based on ASC macrostructure characterization by ns-TEM and NMR analysis on protein–protein interactions (15). In addition, recent comparative studies on the kinetics of ASC and ASCb self-association corroborate PYD and CARD concomitant participation in oligomer formation (16). Interdomain flexibility is required to favor different interactions involving PYD and CARD domains. If we focus firstly on the formation of an ASC dimer, once one domain of a monomer (CARD) has interacted with its homologous in a nearby molecule, the second domain (PYD) will follow. As depicted in Figure 11*C*, this process appears to be relatively simple for ASC, with a linker that allows both the required flexibility and sufficient proximity of the other interacting domain. Thus, the PYD and CARD of ASC readily interact to form oligomers with integral participation of both domains. In this case, intermediate oligomers are not observed experimentally, suggesting that they are short-lived likely leading to extensive polymerization into filaments.

In contrast, dimer formation with simultaneous participation of both PYD and CARD domains is more difficult for ASC3X, as shown in Figure 11*A*. The PYDs of two ASC3X molecules bound through the CARD will need to find each other for dimerization and high-order oligomer formation due to the extra-long linker. Such behavior explains the large remaining NMR signal intensity and the presence of long-lived low-order oligomeric species based on the SEC results. Oligomers that do not readily polymerize would also lead to higher polydispersity, as observed by DLS. All in all, the comparison of the self-association behavior of the natural isoforms ASC and ASCb (16) to that of the engineered adapter ASC3X shown here, together with the detailed study on the interdomain dynamics of the three isoforms, suggest that ASC has an optimal linker for self-association because it confers the needed flexibility while keeping both domains sufficiently close. The differential self-association observed for ASC, ASCb, and ASC3X indicates that the length of the linker can control the function of multidomain proteins.

## Experimental procedures

### Expression and purification of unlabeled and isotopically labeled ASC3X

The expression and purification of ASC3X, ASCb, and ASC were done following previously published protocols (16, 34, 35). Essentially, the purification of the three proteins is done under denaturing conditions *via* His-tag and Ni^2+^ affinity and the proteins are refolded upon denaturant removal.

### Structure of ASC and model of ASC3X

A model of the three-dimensional structure of ASC3X was created based on the structure of ASC (Protein Data Bank 2KN6) (35) using the program Chimera (57). The 23 amino acid linker was tripled to make the 69 amino acid linker in ASC3X.

### Real-time NMR experiments

Real-time NMR experiments performed on ASC3X samples, as well as NMR data processing and analysis followed previously described procedures (16).

### Chemical shift assignment of ASC3X by NMR spectroscopy

ASC3X NMR samples were prepared at 200 μM in 300 μl containing 5% D_2_O, 20 mM glycine, and 1 mM tris(2-carboxyethyl)phosphine at pH 3.8. Backbone chemical shift assignments of ASC3X were obtained from 2D [^1^H-^15^N]-HSQC, 3D HNCACB, and 3D CBCA(CO)NH (58, 59) that were acquired at 298 K on a Bruker Avance III spectrometer operating at a ^1^H frequency of 601.13 MHz and equipped with a cryoprobe. Data were processed with NMRPipe and spectra were analyzed with Sparky (60, 61).

### Backbone amide ^15^N relaxation measurements by NMR

ASC, ASCb, and ASC3X samples at 200 μM concentration were prepared in 20 mM glycine buffer containing 1 mM tris(2-carboxyethyl)phosphine, pH 3.8, and 10 % D_2_O/H_2_O. Relaxation experiments were performed at 298 K. The ^15^N T_1_, T_1ρ_, and {^1^H}-^15^N-NOE data were obtained with specific NMR pulse sequences (44, 46, 62). The recycle delay to measure ^15^N T_1_ and {^1^H}-^15^N-NOE was 3 s, whereas 1.2 s were used for T_1ρ_ experiments. Experiments were acquired in an interleaved manner to minimize effects caused by spectrometer drift. Relaxation measurements of T_1_ and T_1ρ_ were taken at eight different relaxation delays: Δ = 23.987 ms, 103.843 ms, 263.555 ms, 503.123 ms, 762.655 ms, 1002.223 ms, 1301.683 ms, and 1601.143 ms for T_1_ and Δ = 9.524 ms, 19.604 ms, 39.764 ms, 59.444 ms, 79.604 ms, 99.764 ms, 119.924 ms, and 139.604 ms for T_1ρ_. A ^15^N continuous spin-lock field of 2.5 kHz was used for T_1ρ_ experiments. T_2_ values were corrected for off-resonance field using the following equation:

(3) $$1/{\text{T}}_{2}=\;1/\left({\text{T}}_{1\rho }\text{⋅}{\text{sin}}^{2}\;\theta \right)\;-\;1\text{/}\left({\text{T}}_{1}\text{⋅}{\text{tan}}^{2}\;\theta \right)$$

with *tan θ = ω*_*N*_*/Ω*_*N*_, where Ω_N_ is the resonance offset and *ω*_*N*_ is the strength of the spin-lock field.

Relaxation times were calculated by fitting peak-intensity dependence with the experiment relaxation times to an exponential function given by *I(t) = I*_*0*_ *e*^*[(-1/T)t]*^ (T = T_1_, T_1ρ_). T_1_ and T_1ρ_ values are averages of two separate measurements. The {^1^H}-^15^N-NOE values were calculated from the ratio of peak intensities obtained from experiments performed with and without ^1^H presaturation. The ^1^H frequency was shifted off-resonance in the unsaturated experiments. ^1^H presaturation was achieved by a train of 120˚ pulses separated by 5 ms delays and was applied for a total of 3 s. The recycle time is reasonably long; however, NOE values were corrected for incomplete ^1^H magnetization recovery as previously described (62).

Relaxation data were analyzed using the Lipari-Szabo model-free approach (47), which assumes that the overall and internal motions of the protein are independent. The total (*C*), overall (*C*_*o*_), and internal (*C*_*i*_) correlation functions are given by:

(4) $$C\left(t\right)=\;C_{0}\left(t\right)C_{i}\left(t\right)\;$$

(5) $$C_{0}\left(t\right)=\frac{1}{5}e^{\left(-\frac{t}{\text{τ}c}\right)}$$

(6) $$C_{i}\left(t\right)=\;S^{2}+\left(1-S^{2}\right)\cdot e^{\left(-\frac{t}{\text{τ}e}\right)}$$

Where S^2^ is a generalized order parameter describing the amplitude of motion of each N-H bond vector, τ_c_ is the protein’s correlation time, and τ_e_ is the effective correlation time. Under these assumptions, the spectral density function is described as follows:

(7) $$J\left(w\right)=\frac{2}{5}\left(\frac{S^{2}\tau_{c}}{1+{\left(\omega \tau_{c}\right)}^{2}}+\frac{\left(1-S^{2}\right)\tau }{1+{\left(\omega \tau \right)}^{2}}\right)$$

(8) $$\text{where}\;\frac{1}{\tau }=\frac{1}{\tau_{c}}+\frac{1}{\tau_{e}}$$

Apparent rotational correlation times (τ_c_) were obtained from T_1_/T_2_ ratios, which are independent of S^2^ and τ_e_ values for amino acids with heteronuclear NOE values > 0.6 and without significant effects on T_2_ from chemical exchange (45, 63).

### SEC

ASC3X sample preparation for size exclusion chromatographic analysis was done following previously described procedures (16).

### DLS

DLS experiments and ASC3X sample preparation followed previously described procedures (16).

### TEM

ASC3X protein filaments were prepared as previously described (16). Images of ASC3X filaments were obtained using a Talos F200C G2 transmission electron microscope equipped with a Field Emission Gun (X-FEG) at 200 kV. Analysis of ASC3X filaments was performed as previously described (16).

### Mass spectrometry

Mass spectrometry experiments on ASC3X were performed as previously reported (16) with slight modifications. Briefly, NMR samples of ^15^N-ASC3X at ∼700 μM were injected in an electrospray ionization mass spectrometer (Q-Exactive Hybrid Quadrupole-Orbitrap, Thermo). Protein samples were treated as follows: (1) dilution with a solution containing 95% acetonitrile, 4.9% water, 0.1% formic acid, and injection to reverse phase column (Acclaim 200 C18, 3 μm, Thermo operating at a flowrate of 0.3 ml/min) and subsequent mass spectrometer analysis; (2) dilution with 50% acetonitrile and direct injection to mass spectrometer. The spectra were analyzed as previously described (16).

### Molecular docking

The program HADDOCK (52) was used for molecular docking calculations. The ASC3X dimer model was built based on the modeled structure with Chimera (57). HADDOCK was run by selecting as active residues those involved in Death Domain type I interactions (helices H1 and H4 in the CARD of one protomer and helices H2 and H3 in the CARD of the other protomer) (54, 55). Subsequent addition of ASC3X molecules was performed in a similar manner until the formation of the ASC3X 5-member oligomer. In all HADDOCK runs the N- and C-termini were considered charged and the 69 amino acid linker was set to be fully flexible. Each HADDOCK run started with 1,000 structures, and 200 out of which were considered for refinement. Water was used as solvent in each iteration. Structures were chosen based on best the HADDOCK score. ChimeraX was used to analyze ASC3X dimer and pentamer structures (64).

## Data availability

The data that support the findings of this study are contained within the article and the supporting information. All source data generated for this study are available from the corresponding author (Dr Eva de Alba; edealbabastarrechea@ucmerced.edu) upon reasonable request.

## Supporting information

This article contains supporting information.

## Conflict of interest

The authors declare that they have no conflicts of interest with the contents of this article.
