# Supplementary material for: Natural and engineered inflammasome adapter proteins reveal optimum linker length for self-assembly
Source: J Biol Chem. 2022 Sep 16;298(11):102501. doi: 10.1016/j.jbc.2022.102501 (PMC9640978; doi:10.1016/j.jbc.2022.102501)
Supplement: Supporting information [file mmc1.docx]

# Supporting Information:

**­Natural and engineered inflammasome adapter proteins reveal**

**optimum linker length for self-assembly**

Pedro Diaz-Parga^1,2^, Andrea Gould^1,3^ and Eva de Alba^1^

^1^ Department of Bioengineering, School of Engineering, University of California Merced, CA, USA.

^2^ Quantitative Systems Biology Ph.D. Program, University of California Merced, CA, USA.

^3^ Current address: Revolution Medicines. 700 Saginaw Dr. Redwood City, CA, USA.

Corresponding author: Eva de Alba. Department of Bioengineering, School of Engineering, University of California Merced. CA, USA.

**ASC:**

MGSSHHHHHH SSGLVPRGSH

10 20 30 40 50 60
MGRARDAILD ALENLTAEEL KKFKLKLLSV PLREGYGRIP RGALLSMDAL DLTDKLVSFY

 70 80 90 100 110 120
LETYGAELTA NVLRDMGLQE MAGQLQAAT**H QGSGAAPAGI QAPPQSAAKP** **GL**HFIDQHRA

 130 140 150 160 170 180
ALIARVTNVE WLLDALYGKV LTDEQYQAVR AEPTNPSKMR KLFSFTPAWN WTCKDLLLQA

 190
LRESQSYLVE DLERS

**ASCb:**

MGSSHHHHHH SSGLVPRGSH

10 20 30 40 50 60
MGRARDAILD ALENLTAEEL KKFKLKLLSV PLREGYGRIP RGALLSMDAL DLTDKLVSFY

 70 80 90 100 110 120
LETYGAELTA NVLRDMGLQE MAGQLQAAT**H QGL**HFIDQHR AALIARVTNV EWLLDALYGK

 130 140 150 160 170
VLTDEQYQAV RAEPTNPSKM RKLFSFTPAW NWTCKDLLLQ ALRESQSYLV EDLERS

**ASC3X:**

MGSSHHHHHH SSGLVPRGSH

10 20 30 40 50 60
MGRARDAILD ALENLTAEEL KKFKLKLLSV PLREGYGRIP RGALLSMDAL DLTDKLVSFY

 70 80 90 100 110 120
LETYGAELTA NVLRDMGLQE MAGQLQAAT**H QGSGAAPAGI** **QAPPQSAAKP GLHQGSGAAP**

 130 140 150 160 170 180
**AGIQAPPQSA AKPGLHQGSG AAPAGIQAPP QSAAKPGL**HF IDQHRAALIA RVTNVEWLLD

 190 200 210 220 230 240
ALYGKVLTDE QYQAVRAEPT NPSKMRKLFS FTPAWNWTCK DLLLQALRES QSYLVEDLERS

***Figure S1. Amino acid sequence of ASC, ASCb and ASC3X.*** *One-letter code amino acid sequence of full-length ASC, ASCb and ASC3X with residue numbering system. The His-tag and connecting linker to the native sequences is shown in red. Isoform linker is show in bold.*

*
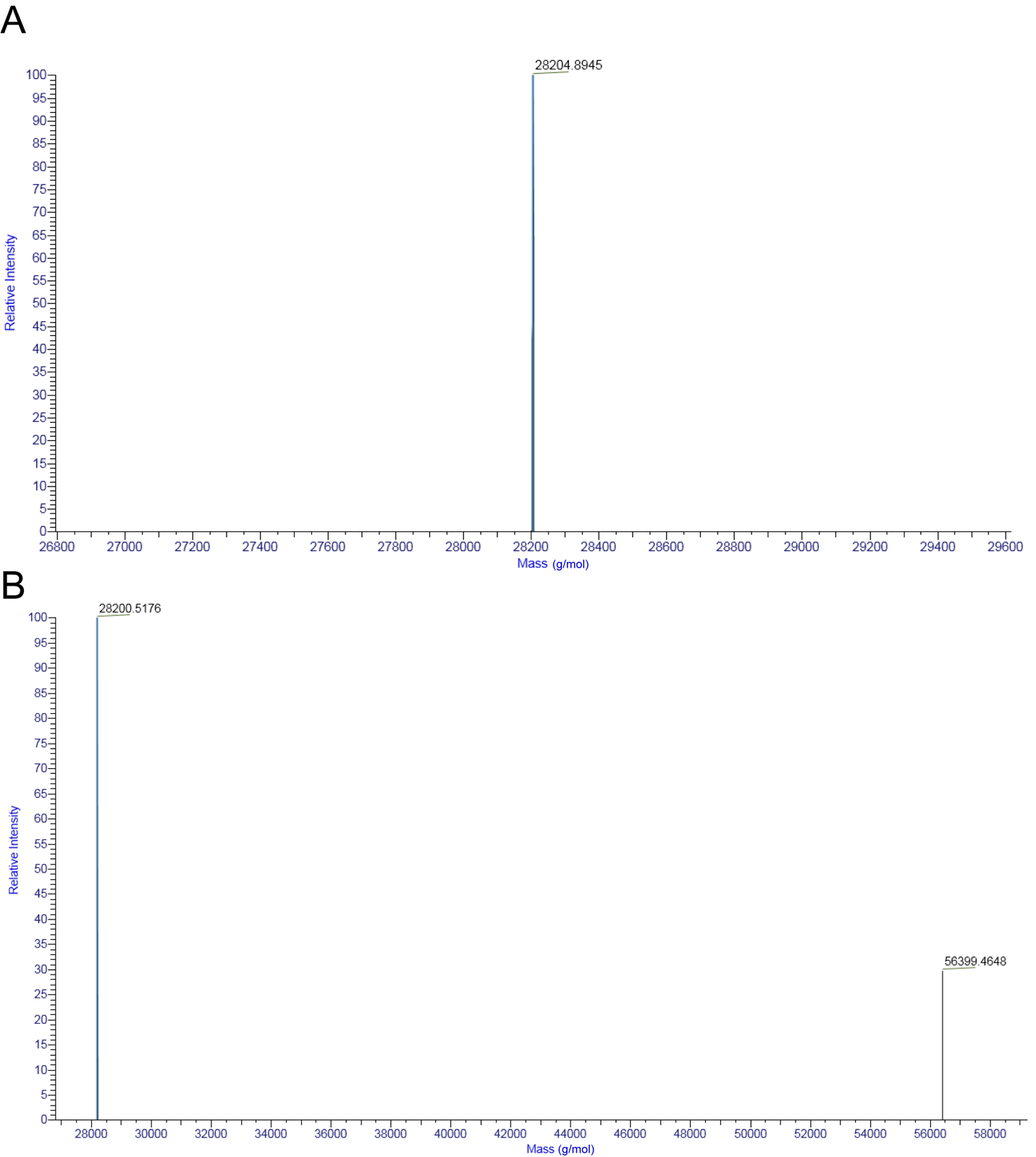
*

***Figure S2. ASC3X monomer and dimer detected by mass spectrometry.*** *Mass spectra of ^15^N-labeled ASC3X in the presence (A) and absence (B) of reverse phase chromatography immediately prior to injection in the mass spectrometer. Spectra were obtained several months after the start of oligomerization.*


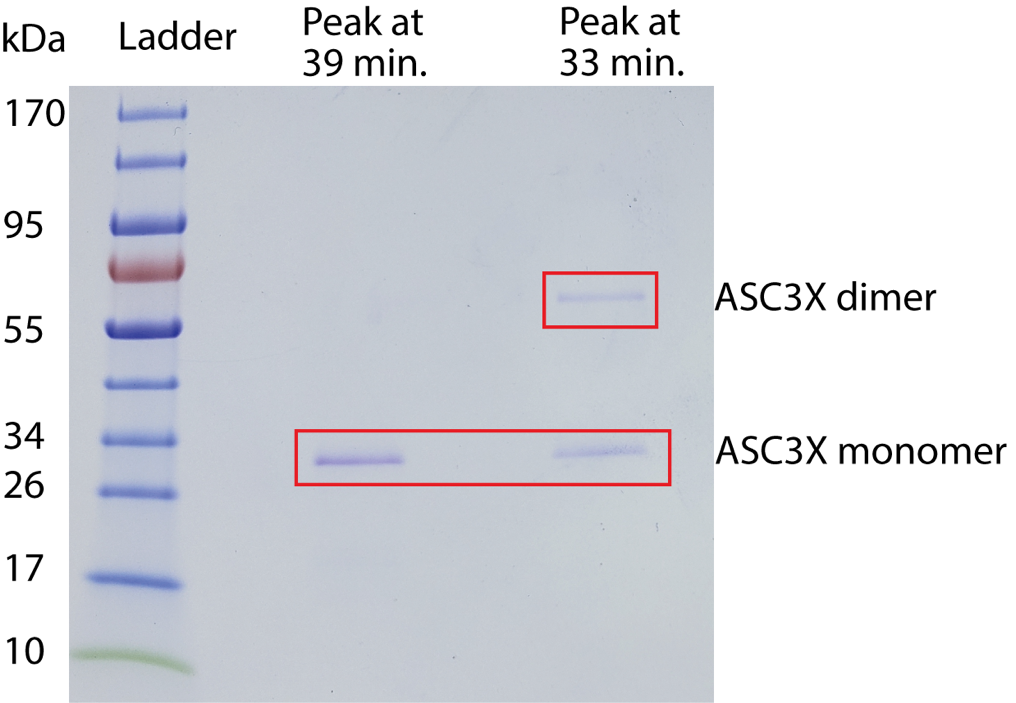


***Figure S3. ASC3X forms stable dimers.*** *SDS-PAGE of ASC3X solutions eluted at 39 and 33 minutes from SEC experiment at 50 μM initial protein concentration, pH 3.8 and 150 mM NaCl.* *ASC3X monomer and dimer are observed in the peak eluted at 33 minutes.*

# Table S1: ^15^N, ^1^HN, ^13^Cα, and ^13^Cβ Chemical Shifts of ASC3X

# (Biomagnetic Resonance Bank ID 51509)

| Residue | ^15^N (ppm) | ^15^NH (ppm) | ^13^C_α_ (ppm) | ^13^C_β_ (ppm) |
| --- | --- | --- | --- | --- |
| M1 | 120.962 | 8.347 | 55.611 | 33.032 |
| G2 | 110.982 | 8.609 | 45.532 | - |
| R3 | 120.226 | 8.290 | 57.309 | 30.771 |
| A4 | 123.951 | 8.632 | 55.461 | 18.398 |
| R5 | 117.246 | 8.531 | 60.138 | 29.713 |
| D6 | 117.294 | 7.713 | 56.837 | 39.644 |
| A7 | 122.635 | 7.695 | 54.987 | 18.751 |
| I8 | 120.631 | 8.125 | 66.243 | 38.811 |
| L9 | 119.737 | 7.945 | 57.758 | 41.657 |
| D10 | 115.688 | 7.991 | 56.275 | 39.248 |
| A11 | 120.081 | 7.387 | 54.795 | 18.131 |
| L12 | 114.735 | 8.266 | 57.695 | 41.28 |
| E13 | 116.465 | 8.802 | 57.279 | 28.1 |
| N14 | 116.130 | 7.312 | 53.501 | 39.864 |
| L15 | 119.411 | 6.912 | 54.453 | 41.265 |
| T16 | 113.064 | 8.223 | 60.96 | 70.61 |
| A17 | 123.426 | 8.929 | 55.836 | 17.858 |
| E18 | 117.576 | 8.587 | 59.13 | 29.032 |
| E19 | 119.474 | 7.680 | 58.441 | 29.931 |
| L20 | 121.935 | 9.055 | 57.908 | 40.63 |
| K21 | 118.623 | 7.528 | 60.168 | 32.231 |
| K22 | 118.617 | 7.602 | 59.792 | 32.638 |
| F23 | 122.143 | 8.796 | 61.461 | 39.533 |
| K24 | 117.308 | 8.081 | 60.555 | 33.355 |
| L25 | 115.859 | 7.715 | 57.813 | 41.314 |
| K26 | 120.631 | 8.125 | 57.915 | 31.981 |
| L27 | 120.799 | 7.913 | 57.279 | 41.233 |
| L28 | 116.130 | 7.312 | 56.09 | 41.748 |
| S29 | 111.009 | 7.455 | 57.997 | 65.397 |
| V30 | 124.079 | 7.846 | - | - |
| L32 | 122.652 | 8.235 | 52.693 | 46.18 |
| R33 | 121.688 | 7.602 | 56.458 | 31.631 |
| E34 | 122.240 | 8.479 | 57.778 | 28.662 |
| G35 | 111.494 | 8.700 | 44.774 | - |
| Y36 | 118.080 | 7.428 | 58.124 | 40.744 |
| G37 | 108.980 | 8.970 | 43.581 | - |
| R38 | 118.392 | 8.482 | 53.835 | 31.468 |
| I39 | 128.499 | 8.217 | - | - |
| R41 | 124.498 | 8.370 | 60.083 | 30.665 |
| G42 | 104.805 | 8.777 | 46.747 | - |
| A43 | 121.883 | 7.189 | 53.547 | 18.492 |
| L44 | 117.047 | 7.808 | 57.204 | 43.14 |
| L45 | 116.164 | 7.798 | 58.829 | 41.251 |
| S46 | 112.031 | 7.674 | 58.762 | 63.905 |
| M47 | 120.099 | 7.204 | 57.874 | 34.655 |
| D48 | 121.942 | 8.706 | 51.104 | 38.929 |
| A49 | 118.611 | 8.574 | 56.02 | 18.329 |
| L50 | 121.570 | 7.881 | 58.536 | 41.41 |
| D51 | 118.608 | 8.205 | 56.986 | 39.432 |
| L52 | 120.694 | 8.705 | 57.522 | 41.905 |
| T53 | 117.975 | 7.979 | 68.027 | 68.027 |
| D54 | 118.176 | 7.714 | 56.947 | 39.103 |
| K55 | 122.730 | 8.332 | 57.456 | 31.517 |
| L56 | 120.629 | 8.957 | 58.846 | 42.642 |
| V57 | 115.229 | 7.963 | 65.801 | 31.322 |
| S58 | 116.728 | 8.192 | 61.666 | 63.643 |
| F59 | 119.967 | 8.456 | 60.708 | 39.028 |
| Y60 | 115.753 | 8.033 | 57.86 | 39.048 |
| L61 | 111.977 | 7.389 | 57.173 | 40.63 |
| E62 | 116.097 | 8.533 | 61.117 | 29.816 |
| T63 | 112.202 | 7.933 | 65.723 | 67.716 |
| Y64 | 122.682 | 8.673 | 58.416 | 35.965 |
| G65 | 106.772 | 7.959 | 47.808 | - |
| A66 | 124.224 | 7.715 | 55.507 | 18.373 |
| E67 | 121.039 | 7.701 | 59.214 | 29.415 |
| L68 | 120.893 | 8.908 | 57.685 | 42.033 |
| T69 | 114.434 | 7.488 | 67.831 | 67.831 |
| A70 | 122.167 | 8.064 | 55.89 | 17.936 |
| N71 | 117.519 | 8.320 | 55.952 | 37.745 |
| V72 | 121.974 | 8.407 | 67.177 | 30.961 |
| L73 | 118.545 | 8.341 | 58.284 | 41.224 |
| R74 | 119.130 | 8.419 | 60.776 | 29.419 |
| D75 | 121.787 | 8.100 | 56.336 | 39.679 |
| M76 | 116.607 | 7.852 | 56.663 | 35.047 |
| G77 | 107.353 | 7.936 | 45.321 | - |
| L78 | 124.636 | 8.042 | 52.819 | 38.855 |
| Q79 | 118.782 | 7.807 | 59.729 | 28.071 |
| E80 | 121.151 | 8.737 | 59.324 | 27.695 |
| M81 | 119.810 | 8.048 | 58.93 | 33.496 |
| A82 | 121.434 | 8.047 | 55.387 | 19.3 |
| G83 | 104.981 | 8.258 | 46.942 | - |
| Q84 | 122.257 | 7.917 | 58.465 | 28.245 |
| L85 | 122.425 | 7.656 | 58.367 | 41.693 |
| Q86 | 119.172 | 8.282 | 59.057 | 28.714 |
| A87 | 121.504 | 8.015 | 54.675 | 18.108 |
| A88 | 119.258 | 7.816 | 53.869 | 19.152 |
| T89 | 105.292 | 7.460 | 61.804 | 69.592 |
| H90 | 119.206 | 7.683 | 55.843 | 28.317 |
| Q91 | 120.939 | 8.236 | 55.976 | 29.734 |
| G92 | 110.387 | 8.439 | 45.138 | - |
| L112 | 121.248 | 8.116 | 54.218 | 43.486 |
| H113 | 125.772 | 9.483 | 58.698 | 31.626 |
| F114 | 129.973 | 8.281 | 60.526 | 38.064 |
| I115 | 115.825 | 11.286 | 64.527 | 38.076 |
| D162 | 117.746 | 7.211 | 56.303 | 41.814 |
| Q163 | 119.783 | 8.221 | 57.906 | 29.124 |
| H164 | 110.626 | 7.146 | 53.119 | 28.091 |
| R165 | 121.561 | 6.533 | 60.391 | 30.962 |
| A166 | 118.502 | 8.178 | 55.522 | 17.803 |
| A167 | 121.133 | 7.859 | 54.473 | 19.859 |
| L168 | 116.766 | 8.093 | 57.571 | 41.678 |
| I169 | 118.239 | 8.085 | 65.81 | 38.356 |
| A170 | 115.195 | 7.127 | 53.87 | 20.213 |
| R171 | 112.040 | 7.847 | 56.775 | 34.159 |
| V172 | 121.068 | 7.755 | 64.634 | 31.862 |
| T173 | 116.174 | 8.333 | 60.309 | 70.392 |
| N174 | 119.632 | 8.079 | 52.541 | 37.036 |
| V175 | 118.967 | 7.965 | 65.673 | 31.653 |
| E176 | 119.203 | 8.558 | 60.168 | 28.151 |
| W177 | 118.179 | 7.611 | 61.008 | 29.591 |
| L178 | 116.130 | 7.312 | 57.81 | 41.393 |
| L179 | 115.692 | 8.166 | 57.597 | 40.981 |
| D180 | 117.643 | 8.205 | 56.607 | 38.443 |
| A181 | 121.688 | 7.602 | 54.046 | 18.074 |
| L182 | 114.330 | 7.413 | 54.739 | 43.272 |
| Y183 | 126.314 | 7.980 | 60.728 | 37.713 |
| G184 | 120.008 | 8.409 | 46.151 | - |
| K185 | 120.123 | 7.473 | 57.767 | 33.76 |
| V186 | 112.576 | 7.478 | 63.139 | 34.457 |
| L187 | 117.267 | 7.212 | 53.51 | 44.617 |
| T188 | 113.142 | 8.032 | 60.22 | 70.596 |
| D189 | 120.334 | 8.929 | 58.479 | 40.611 |
| E190 | 117.274 | 8.562 | 59.644 | 28.431 |
| Q191 | 119.760 | 7.359 | 58.245 | 29.166 |
| Y192 | 119.203 | 8.558 | 61.883 | 39.392 |
| Q193 | 115.820 | 8.554 | 58.291 | 27.873 |
| A194 | 121.208 | 7.872 | 54.802 | 18.564 |
| V195 | 116.466 | 7.947 | 66.643 | 31.256 |
| R196 | 117.694 | 7.842 | 59.341 | 29.956 |
| A197 | 116.396 | 7.151 | 52.802 | 19.112 |
| E198 | 119.360 | 7.487 | - | - |
| T200 | 106.501 | 7.091 | 58.404 | 73.142 |
| N201 | 120.764 | 9.526 | - | - |
| S203 | 111.619 | 7.338 | 62.538 | 62.538 |
| K204 | 125.309 | 8.680 | 60.723 | 35.03 |
| M205 | 116.349 | 8.498 | 55.492 | 30.921 |
| R206 | 120.558 | 8.791 | 60.426 | 30.35 |
| K207 | 120.108 | 7.639 | 57.015 | 30.977 |
| L208 | 122.988 | 8.572 | 58.403 | 41.622 |
| F209 | 114.057 | 8.435 | 60.609 | 37.633 |
| S210 | 118.416 | 7.659 | 60.845 | 62.474 |
| F211 | 119.877 | 7.135 | 56.521 | 38.625 |
| T212 | 110.180 | 6.839 | - | - |
| A214 | 115.195 | 7.127 | 51.699 | 19.452 |
| W215 | 120.631 | 8.125 | 55.379 | 30.954 |
| N216 | 118.913 | 8.102 | 51.21 | 38.732 |
| W217 | 120.812 | 8.752 | 61.446 | 29.207 |
| T218 | 113.106 | 7.852 | 66.537 | 68.584 |
| C219 | 119.691 | 8.010 | 63.812 | 26.651 |
| K220 | 118.734 | 7.582 | 61.315 | 29.089 |
| D221 | 119.904 | 8.521 | 57.139 | 39.486 |
| L222 | 122.909 | 7.811 | 58.112 | 41.107 |
| L223 | 120.617 | 7.453 | 58.448 | 40.718 |
| L224 | 120.495 | 8.173 | 58.301 | 42.785 |
| Q225 | 119.405 | 8.678 | 58.846 | 28.1 |
| A226 | 121.462 | 7.824 | 55 | 18.119 |
| L227 | 121.288 | 8.471 | 57.749 | 42.363 |
| R228 | 119.351 | 8.516 | 60.135 | 29.911 |
| E229 | 114.624 | 7.720 | 57.843 | 29.295 |
| S230 | 110.396 | 7.492 | 59.075 | 64.772 |
| Q231 | 124.464 | 8.568 | 54.494 | 29.095 |
| S232 | 117.948 | 8.407 | 62.3 | 62.3 |
| Y233 | 118.782 | 7.807 | 59.712 | 36.909 |
| L234 | 122.879 | 6.936 | 57.235 | 41.401 |
| V235 | 116.919 | 7.151 | 67.356 | 31.361 |
| E236 | 116.651 | 8.043 | 58.785 | 28.277 |
| D237 | 118.328 | 7.901 | 56.745 | 40.635 |
| L238 | 118.069 | 7.890 | 56.422 | 41.905 |
| E239 | 116.943 | 7.999 | 57.373 | 29.096 |
| R240 | 118.133 | 7.321 | 56.539 | 30.678 |
| S241 | 120.791 | 7.672 | - | - |
